# Layer‐Specific Dermal and Subcutaneous Delivery via Modular LNP–Hydrogel‐Integrated Threaded Microneedles

**DOI:** 10.1002/advs.77624

**Published:** 2026-09-21

**Authors:** Fangzhou Xie, Yixiang Zhang, Shifeng Ling, Yuxuan Huang, Yingying Wei, Rao Fu, Jiahao He, Baikun Liu, Lucia Zhou, Kaifeng Huo, Yida Wang, Qingfeng Li, Yawei Du, Wenguo Cui, Yun Xie

**Affiliations:** ^1^ Department of Plastic & Reconstructive Surgery Shanghai Ninth People's Hospital Shanghai Jiao Tong University School of Medicine Shanghai People's Republic of China; ^2^ Department of Plastic Surgery, State Key Laboratory of Trauma, Burns and Combined Injury Southwest Hospital, Third Military Medical University (Army Medical University) Chongqing People's Republic of China; ^3^ Department of Orthopaedics, Shanghai Key Laboratory For Prevention and Treatment of Bone and Joint Diseases, Shanghai Institute of Traumatology and Orthopaedics Ruijin Hospital, Shanghai Jiao Tong University School of Medicine Shanghai People's Republic of China; ^4^ Shanghai Jiao Tong University School of Medicine Shanghai People's Republic of China

**Keywords:** layer‐specific delivery, lipid nanoparticles, microneedles, mRNA therapeutics

## Abstract

Skin diseases exhibit hierarchical pathological remodeling across the epidermis, dermis, and subcutaneous tissue. However, current therapies lack precise and controllable layer‐specific intervention. Here, we present a threaded microneedle‐mRNA platform enabling stratified, targeted delivery. Guided by spatial transcriptomics and clinical validation, we identified layer‐specific targets in skin fibrosis. Optimized lipid nanoparticles (LNPs) were integrated into a microneedle design featuring a fast‐releasing superficial hydrogel that delivers a SIRT1 agonist to mitigate dermal fibroblast senescence, and helical grooves that directionally deposit PGC1α mRNA–LNPs into subcutaneous adipose during rotational insertion for in situ expression and reversal of adipocyte fibrotic phenotypes. In bleomycin‐induced murine fibrosis, the system reduced dermal senescence and ECM deposition, restored subcutaneous adipose architecture, and attenuated overall fibrosis. This layer‐specific microneedle–mRNA strategy offers a precise therapeutic pathway for complex skin diseases.

## Graphical abstract

Guided by spatial transcriptomics and clinical validation, a threaded microneedle platform enables precise layer‐specific delivery across the dermis and subcutaneous tissue. By addressing distinct pathogenic remodeling in different skin layers, this strategy coordinates compartment‐specific intervention, restores tissue architecture, and offers a precise therapeutic approach for skin fibrosis.

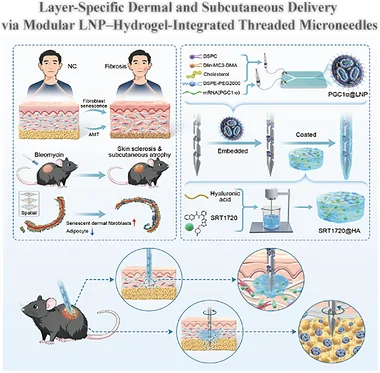

## 1 Introduction

The onset and recurrence of skin diseases are closely associated with abnormal remodeling across distinct skin layers, including the epidermis, dermis, and subcutaneous tissue; however, current therapeutic strategies lack effective layer‐specific interventions [1, 2]. Physiologically, the epidermis is predominantly composed of keratinocytes and functions as a primary barrier and immune surveillance interface [3, 4]. The dermis is enriched with fibroblasts and vascular networks, providing mechanical support for tissue repair [5]. The subcutaneous layer mainly consists of adipocytes and stromal cells, serving as a reservoir for energy metabolism and regenerative capacity [6, 7]. Recent advances in single‐cell and spatial transcriptomics have demonstrated that skin pathological responses exhibit pronounced layer‐specific patterns [8, 9]. Specifically, the epidermal–dermal junction is characterized by inflammatory cell infiltration and barrier disruption; the reticular dermis is dominated by fibroblast activation, myofibroblast differentiation, and excessive extracellular matrix (ECM) deposition leading to fibrosis; whereas the subcutaneous layer exhibits adipose atrophy, metabolic dysregulation, and remodeling of the immune microenvironment [10, 11, 12, 13]. Consequently, precise and coordinated layer‐specific interventions targeting distinct skin compartments have emerged as a critical strategy to enhance therapeutic efficacy and reduce disease recurrence. Integrating multi‐omics–driven target identification with the programmable and rapidly validated mRNA platform may provide a transformative pathway for drug development in skin diseases, ultimately enabling precise therapies tailored to specific skin layers.

Currently, mRNA‐based therapies have begun to be explored in cutaneous applications, particularly in wound healing [14, 15]. For example, VEGF‐A mRNA has been shown to promote angiogenesis and tissue repair in diabetic wounds [16]. However, efficient and mature mRNA delivery technologies for skin applications remain lacking. Existing approaches face substantial challenges in traversing the stratum corneum and fail to achieve layer‐specific interventions within target skin compartments [17]. Conventional delivery methods, such as subcutaneous injection or electroporation, lead to predominant retention of mRNA within the superficial dermis, resulting in insufficient local expression and limited control over delivery depth [18, 19]. In parallel, microneedle patches have emerged as a promising strategy for transdermal delivery, offering minimally invasive penetration of the stratum corneum together with controllable insertion depth and spatial distribution [20, 21, 22, 23]. By modularly integrating mRNA delivery vehicles with polymeric matrices within microneedle systems, it may be possible to achieve efficient, localized, and programmable mRNA expression while maintaining minimally invasive delivery [24, 25]. On this basis, further development of microneedle platforms with layer‐specific intervention capability is expected to better accommodate the hierarchical pathology of diverse skin diseases, and to advance precise and durable in situ therapies.

Acupuncture‐based microneedles allow fine control of millimeter‐scale insertion depth for single needles or small clusters through manual manipulation or mechanical depth limiters [26]. Compared with microneedle patches, they offer superior operability and target‐layer controllability for small‐ to medium‐sized lesions, particularly in irregular anatomical regions such as curved surfaces, periarticular areas, and skin folds [27]. However, the drug delivery capacity of conventional acupuncture microneedles remains limited. Drug loading typically relies on surface coating or soaking, resulting in most cargos being retained within the stratum corneum and superficial epidermis, which hinders efficient traversal of the epidermal–dermal barrier and deposition into target dermal layers. To enable deep and three‐dimensional delivery along the needle insertion pathway, our group previously developed a threaded microneedle platform [28, 29]. The needle surface is engineered with continuous helical grooves and a controllable rotational insertion mechanism, providing stable propulsion and a consistent tissue–needle friction interface during penetration. These grooves accommodate, protect, and guide nanoformulated cargos to undergo layer‐resolved deposition along the insertion trajectory, thereby reducing shear stress and tissue damage while enhancing delivery stability and reproducibility [30]. The platform has demonstrated favorable biocompatibility and delivery efficiency across multiple models. In neural repair, threaded microneedles enabled directional deposition and sustained release of neurotrophic factors, promoting axonal regeneration and synaptic reconstruction [31]. In cartilage repair, groove‐assisted delivery significantly increased penetration depth into the subchondral region, facilitating matrix regeneration and restoration of mechanical properties [32]. Collectively, leveraging a threaded microneedle platform to integrate mRNA technologies holds substantial potential for developing layer‐specific therapies for skin diseases.

**SCHEME 1:**
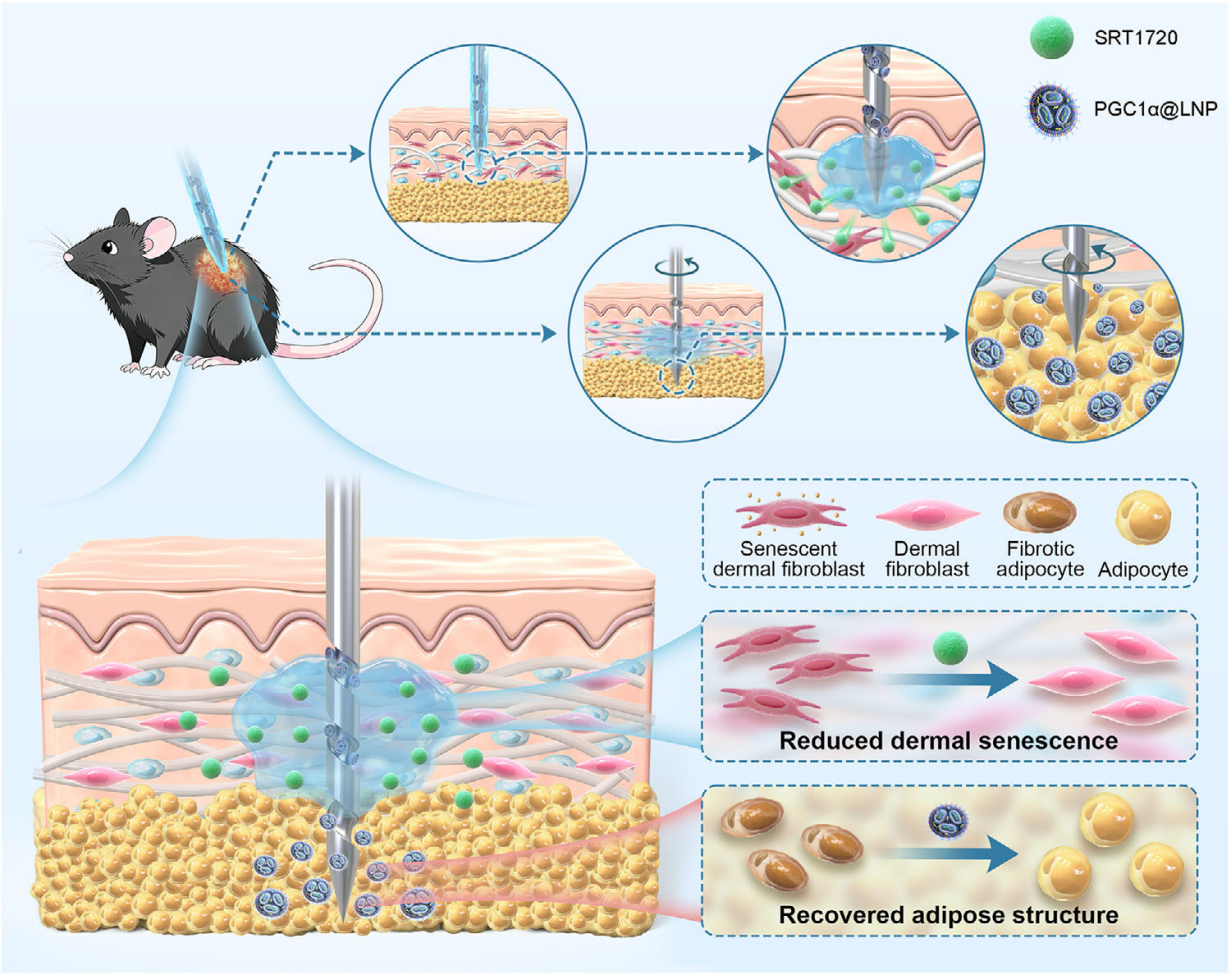
Layer‐specific dermal/subcutaneous mRNA delivery using the LNP–hydrogel integrated threaded microneedle. During its advancement through the dermis, the HA‐based coating loaded with SRT1720@HA is released into the dermal layer, leading to reduced dermal senescence. Upon reaching the subcutaneous tissue, rotation‐driven discharge from the spiral grooves enables the targeted release of PGC1α@LNP, resulting in recovered adipose structure within the subcutaneous layer.

Skin fibrosis is a representative layer‐specific remodeling skin disease, characterized primarily by fibroblast activation and excessive extracellular matrix (ECM) deposition in the reticular dermis, with potential extension toward the epidermis and subcutaneous tissue, ultimately leading to structural and functional imbalance [33, 34]. Based on a threaded microneedle platform, this work uses skin fibrosis as a model to develop a layer‐specific microneedle–mRNA therapeutic strategy. By emphasizing deep compatibility with mRNA platforms and layer‐specific delivery design, the approach addresses two major bottlenecks in cutaneous mRNA therapy—the stratum corneum barrier and limited control over insertion depth—thereby enabling directional deposition in the reticular dermis, efficient in situ expression, and programmable layer‐specific intervention. Using an integrated strategy combining spatial transcriptomics and molecular biological validation, we identified layer‐specific therapeutic targets, including SIRT1 in the epidermal/superficial dermal region and PGC‐1α in the dermal–subcutaneous transitional zone [35]. mRNA delivery vehicles compatible with microneedle fabrication were then developed through optimization of lipid nanoparticle (LNP) formulations. An integrated layered delivery scheme was established, in which a rapidly releasing hydrogel coating containing a SIRT1 agonist was applied to the needle surface to suppress the senescence‐associated secretory phenotype (SASP) of senescent dermal fibroblasts [36], while PGC‐1α mRNA‐loaded LNPs were embedded within the helical grooves and directionally deposited into the dermal–subcutaneous compartment during rotational insertion to enable in situ expression and coordinated spatiotemporal intervention. In bleomycin‐induced skin fibrosis mice, treatment resulted in reduced dermal senescence, downregulation of SASP‐associated signaling, restoration of subcutaneous adipose architecture, and a significant overall attenuation of fibrosis. Collectively, this threaded microneedle system enables dermal–subcutaneous layer‐specific delivery of small‐molecule drugs and mRNA, providing a versatile strategy for precise intervention in skin diseases (Scheme 1).

## 2 Results and Discussion

### 2.1 Spatial and Cellular Dissection of Layer‐Specific Pathological Phenotypes in Fibrotic Skin

**FIGURE 1:**
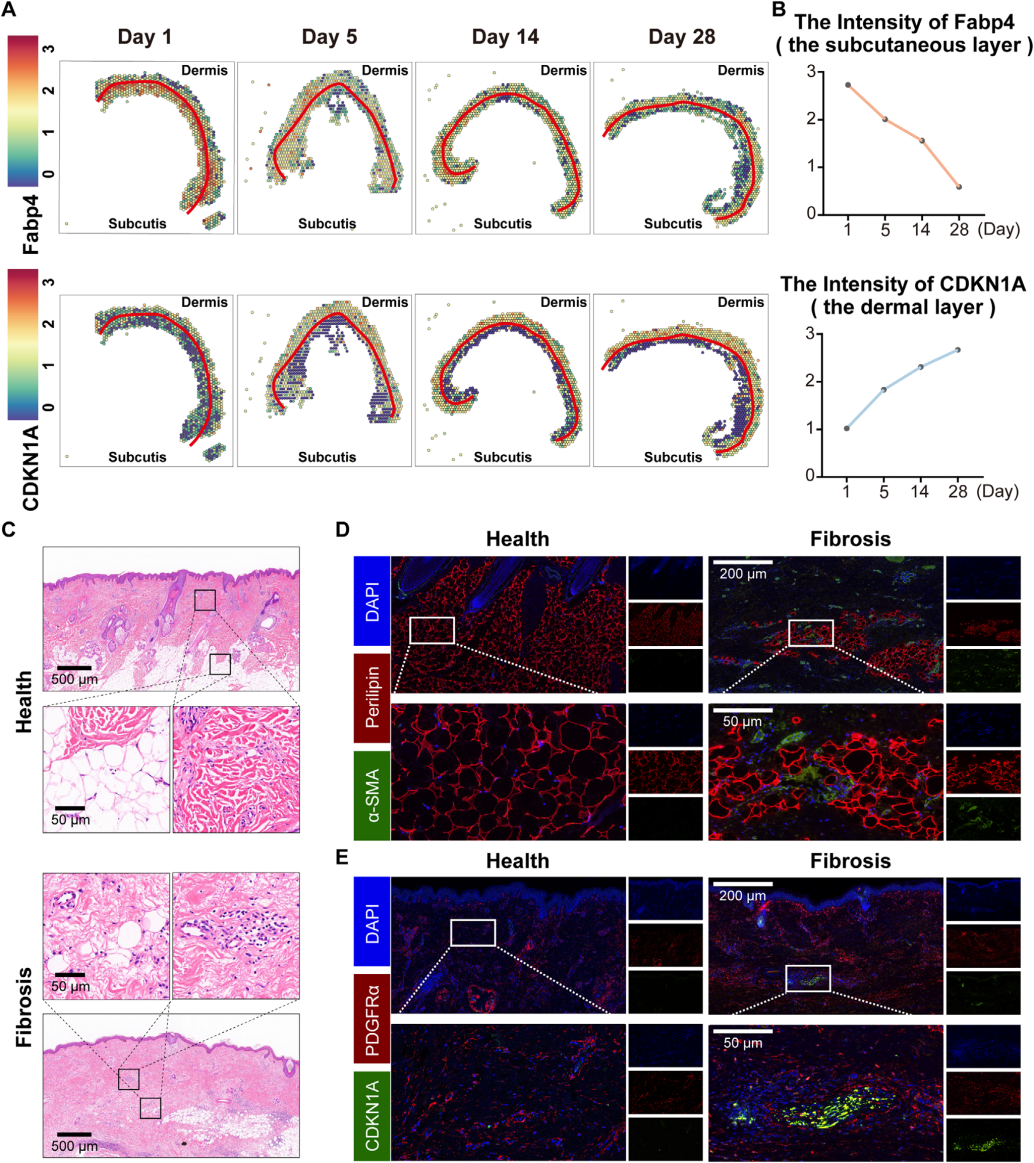
Spatial and cellular characterization of layer‐specific pathological features in skin fibrosis. (A) Spatial transcriptomic maps of bleomycin‐induced fibrotic skin showing the distributions of Fabp4 and CDKN1A in the dermal and subcutaneous compartments. The dermal and subcutaneous layers are labeled, and their boundary is indicated by the red dashed lines. (B) Semi‐quantitative analysis of spatial transcriptomic signals corresponding to panel A, showing layer‐specific expression intensities of Fabp4 and CDKN1A. (C) H&E staining of clinical skin fibrosis specimens depicting dermal collagen architecture and the subdermal adipose compartment. (D) Immunofluorescence staining of subcutaneous tissue with Perilipin and α‐SMA. Nuclei are counterstained with DAPI. (E) Immunofluorescence staining of dermal tissue with CDKN1A and PDGFRα. Nuclei are counterstained with DAPI. Scale bars are shown in each panel unless otherwise specified.

Spatial transcriptomic profiling of bleomycin‐induced fibrotic skin revealed distinct layer‐specific pathological patterns. With continuous bleomycin exposure, the adipocyte marker Fabp4 progressively decreased in the subcutaneous tissue, while the senescence marker Cdkn1a gradually increased within the dermal compartment (Figure 1A,B). HE staining of clinical fibrotic skin specimens showed disorganized dermal collagen fibers and fibrotic replacement of the subdermal adipose layer (Figure 1C). Immunofluorescence analysis further confirmed this stratified remodeling: Perilipin/α‐SMA co‐staining indicated AMT in the subcutaneous layer, and CDKN1A/PDGFRA co‐localization revealed senescent fibroblast accumulation within the dermis (Figure 1D,E).

Similar layer‐specific alterations have been reported in skin fibrosis models, where adipocyte‐derived cells contribute to myofibroblast pools and fat loss, while senescent fibroblasts promote matrix stiffness through persistent SASP signaling and NF‐κB/TGF‐β activation [37, 38, 39, 40]. Building upon these observations, our spatial transcriptomic and histological data reveal a layer‐specific pathological architecture in skin fibrosis, with localized scleroderma serving as a representative fibrotic skin disease characterized by dermal fibroblast senescence and subcutaneous adipocyte transdifferentiation. This spatial pattern suggests that the dermis and subcutis follow distinct pathogenic programs, with the former driven by inflammation and senescence and the latter by metabolic dysfunction and fibrosis, thereby underscoring the necessity of layer‐specific therapeutic strategies.

### 2.2 Structural and Functional Characterization of PGC1α@LNP and SRT1720@HA

**FIGURE 2:**
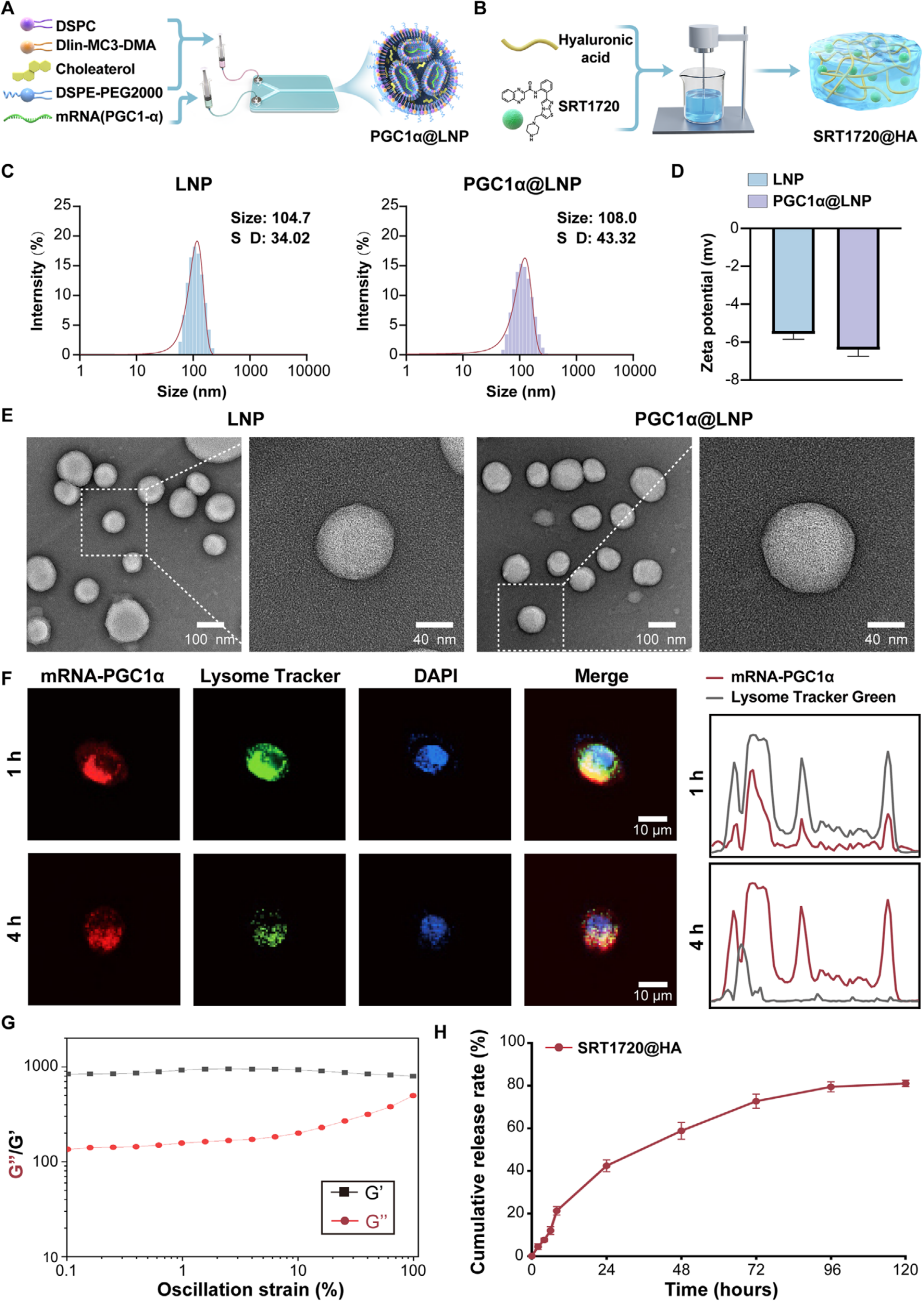
Structural characterization of PGC1α@LNP and SRT1720@HA. (A) Schematic illustration of PGC1α@LNP formulation. (B) Schematic illustration of SRT1720@HA. (C) Particle size distributions of blank LNP and PGC1α@LNP measured by DLS. (D) Zeta potentials of blank LNP and PGC1α@LNP. (E) TEM images showing spherical morphology and uniform particle size of both blank LNP and PGC1α@LNP. (F) Confocal imaging showing reduced colocalization of PGC1α@LNP with lysosomes at 4 h compared with 1 h, indicating time‐dependent endosomal escape. (G) Rheological profiles of SRT1720@HA showing typical viscoelastic solid behavior with Gʹ > Gʺ across strain ranges. (H) In vitro release profile of SRT1720 from the HA hydrogel, showing sustained cumulative release over 96 h. Scale bars are shown in each panel unless otherwise specified.

DLS analysis showed that both blank LNP and PGC1α@LNP exhibited uniform particle size distributions, with mean diameters of 104.7 and 108.0 nm (Figure 2C). Zeta potential measurement revealed that encapsulation of PGC1α slightly decreased the surface potential from –5.2 to –6.4 mV, suggesting successful nucleic acid incorporation without aggregation (Figure 2D). TEM images further confirmed that both formulations maintained well‐defined spherical morphologies with smooth surfaces and homogeneous size distribution (Figure 2E). PGC1α@LNP achieved an encapsulation efficiency of 92.6 ± 2.1%, with an mRNA loading of 18.4 ± 1.2 µg/mg lipid. The formulation exhibited sustained mRNA release, with cumulative release approaching 65% at 96 h (Figure S1A). Confocal imaging showed reduced colocalization of PGC1α@LNP with lysosomes at 4 h compared with 1 h, indicating time‐dependent endosomal escape (Figure 2F). In 3T3‐L1 cells, freshly prepared, lyophilized and immediately reconstituted, and one‐month‐stored PGC1α@LNP formulations all increased PGC1α mRNA and protein expression, confirming effective intracellular delivery and translation of the encapsulated mRNA. The comparable expression levels among the three formulations further indicated that PGC1α@LNP retained its functional stability after lyophilization and one month of storage (Figure S1B,C).

For the hydrogel component, the actual SRT1720 loading was 1.34 ± 0.05 mg/mL hydrogel, corresponding to a loading efficiency of 95.0 ± 3.5%. Rheological analysis showed that SRT1720@HA exhibited typical viscoelastic solid behavior, with the storage modulus (G′) exceeding the loss modulus (G″) across increasing oscillation strain, indicating a stable viscoelastic hydrogel network (Figure 2G). The in vitro drug release profile showed a sustained release pattern of SRT1720 from the HA matrix, reaching approximately 80% cumulative release within 96 h (Figure 2H). Together, these results demonstrate that PGC1α@LNP possesses suitable physicochemical properties and functional stability, while SRT1720@HA provides a stable matrix for sustained drug release.

### 2.3 Structural Characterization of the Threaded Microneedle System

**FIGURE 3:**
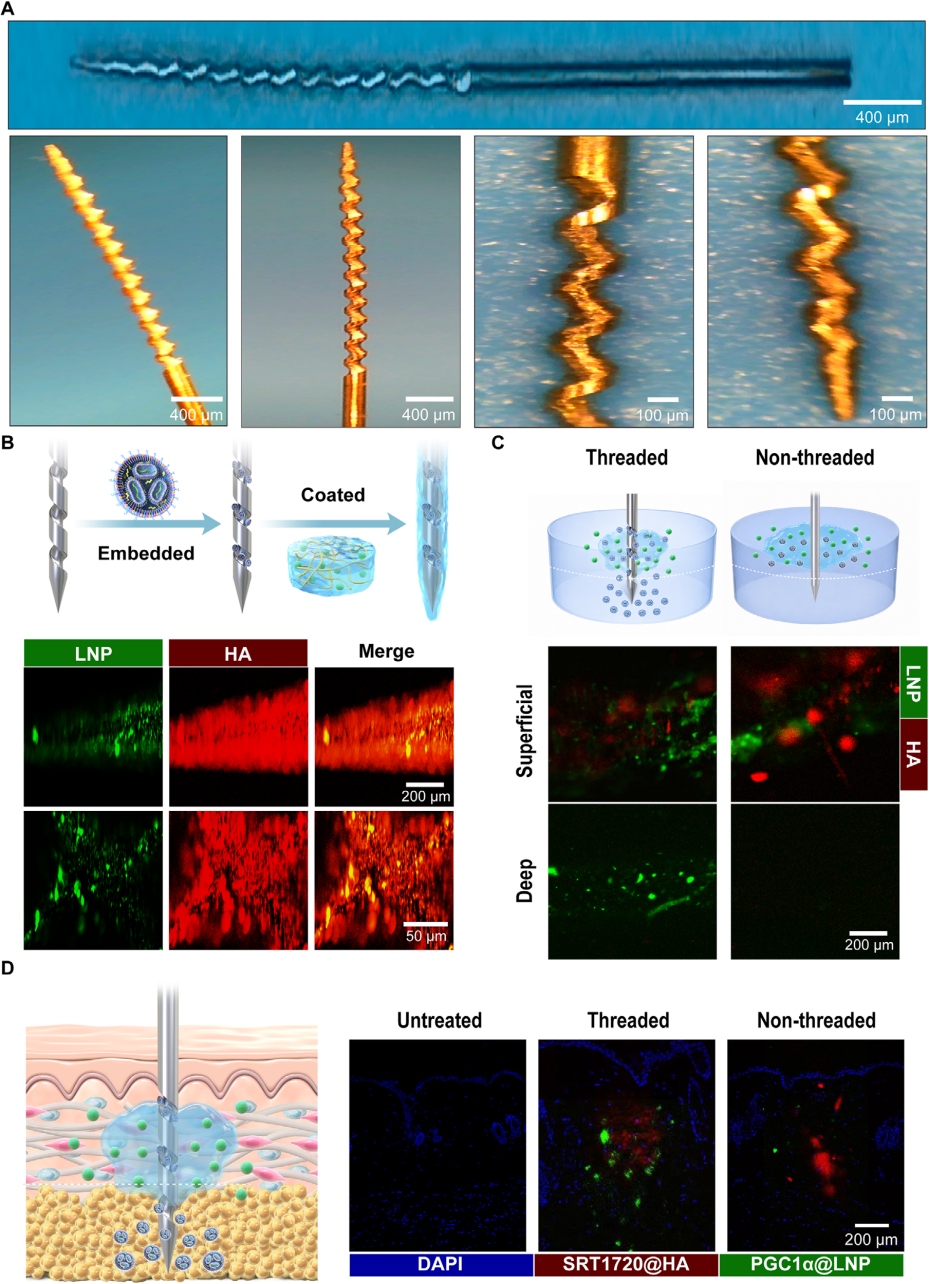
Structural characterization of the threaded microneedle system. (A) Optical microscopy image of the threaded microneedle showing its spiral groove structure. (B) Fluorescence image of the microneedle coated with LNPs (green) and HA hydrogel (red). (C) Cross‐sectional fluorescence image of the coated microneedle inserted into a simulated skin model, showing the spatial distribution of LNPs (green) and HA hydrogel (red). (D) In vivo fluorescence image showing the distribution of PGC1α@LNP (green) and SRT1720@HA (red) in murine skin. Scale bars are shown in each panel unless otherwise specified.

The fabricated threaded microneedle exhibited a distinct spiral groove structure with uniform pitch and consistent surface morphology, as shown under optical microscopy (Figure 3A). Drug loading was quantified using pooled microneedles. Batch‐based quantification showed that the average PGC1α@LNP loading was 2.40 ± 0.23 µg per microneedle, corresponding to a loading efficiency of 80.0 ± 7.5% and approximately 44 ng of encapsulated PGC1α mRNA per microneedle. LC–MS/MS analysis of pooled coated microneedles showed an average SRT1720 loading of 15.1 ± 1.3 ng per microneedle. Fluorescence imaging of the coated microneedles confirmed the distribution of green‐labeled LNPs and red‐labeled SRT1720@HA hydrogel over the designated drug‐loading region. Separate fluorescence channels further verified the spatial distribution of the two components along the microneedle surface (Figure 3B). Threaded and non‐threaded microneedles with the same fluorescent coatings were then inserted to the same depth into a simulated skin model. The threaded microneedle produced deeper, more continuous LNP deposition along the insertion tract, whereas the non‐threaded microneedle showed mainly superficial and localized deposition. HA fluorescence remained concentrated in the upper insertion region in both groups (Figure 3C). In murine skin, the threaded microneedle similarly promoted PGC1α@LNP distribution from the dermis into the subcutaneous layer, while the non‐threaded microneedle produced more limited and superficial distribution. SRT1720@HA was mainly retained in the dermis (Figure 3D). This distribution pattern was attributed to the external localization of the HA hydrogel coating, which promoted its removal and retention within the dermis during insertion [41], whereas the spiral grooves protected the lyophilized LNP formulation from premature displacement and facilitated its transport to greater tissue depths [28, 29, 31]. These results demonstrate that the threaded microneedle system enables differential spatial deposition of the two formulations, characterized by preferential dermal retention of SRT1720@HA and deeper, tract‐wide distribution of PGC1α@LNP.

### 2.4 Biocompatibility Evaluation of SRT1720@HA and PGC1α@LNP

**FIGURE 4:**
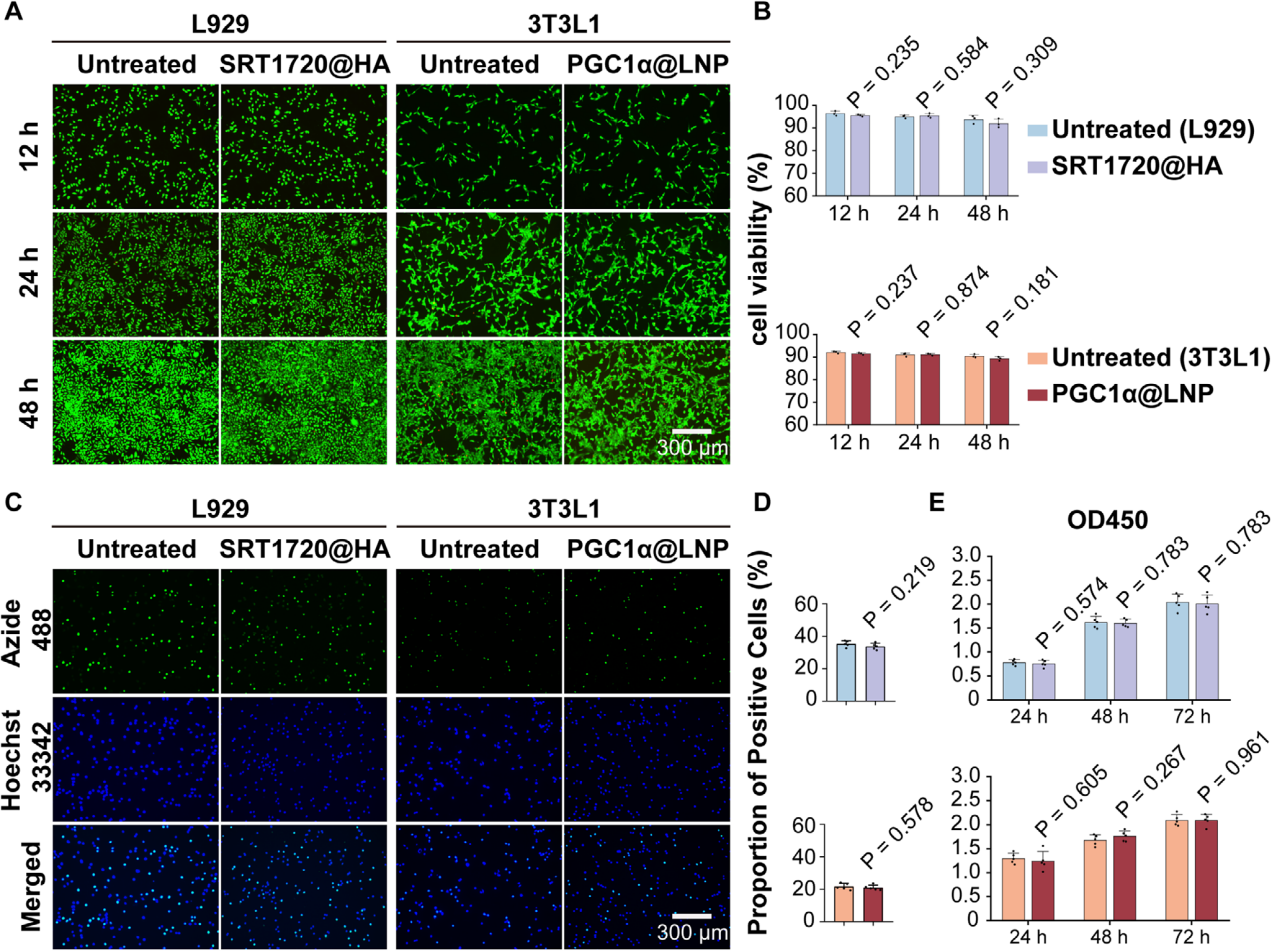
In vitro biocompatibility assessment of SRT1720@HA and PGC1α@LNP. (A) Calcein‐AM/PI staining of L929 fibroblasts and differentiated 3T3‐L1 adipocytes after exposure to SRT1720@HA extract (100% v/v) or PGC1α@LNP (100 µg/mL total lipid) for 12, 24, and 48 h. (B) Quantification images corresponding to the Calcein‐AM/PI staining shown in panel A. (C) EdU staining of L929 fibroblasts and differentiated 3T3‐L1 adipocytes following treatment with SRT1720@HA extract or PGC1α@LNP for 24 h. Nuclei are counterstained with Hoechst. (D) Quantification plots corresponding to the EdU staining shown in panel C. (E) CCK‐8 assay of L929 fibroblasts and differentiated 3T3‐L1 adipocytes treated with SRT1720@HA extract or PGC1α@LNP for 24, 48, and 72 h. All scale bars are shown in the respective panels unless otherwise specified. Statistical analyses were performed using unpaired two‐tailed t‐tests (mean ± SD).

L929 fibroblasts were selected as a surrogate for primary dermal fibroblasts, and adipogenically differentiated 3T3‐L1 cells were used as the canonical model of subcutaneous adipocytes. These lineage‐relevant models were consistent with the dermal–subcutaneous targeting strategy of this study. Cells were exposed to a working level of each carrier—SRT1720@HA extract (100% v/v) or PGC1α@LNP (100 µg/mL total lipid)—and compared with blank‐medium controls. CCK‐8 measurements increased over 24/48/72 h in both treated and control cultures with no between‐group differences (Figure 4E). EdU incorporation at 24 h was unchanged relative to controls (Figure 4C,D), and Calcein‐AM/PI staining at 12/24/48 h showed predominantly Calcein‐positive, PI‐negative monolayers under all conditions (Figure 4A,B). To further assess concentration‐dependent cytocompatibility, cells were exposed for 24 h to SRT1720@HA extracts at 25%, 50%, 75%, and 100% (v/v), or to PGC1α@LNP at total lipid concentrations of 25, 50, 100, and 200 µg/mL. CCK‐8 analysis showed no significant reduction in cell viability across the tested concentration ranges in either cell type. Together, these findings demonstrate that SRT1720@HA and PGC1α@LNP were well tolerated within the tested exposure ranges and did not adversely affect cell viability or basal proliferation (Figure S2).

### 2.5 Systemic Exposure After Local Microneedle Application

To assess tissue distribution and systemic exposure after local microneedle administration, PGC1α mRNA and SRT1720 were examined in plasma, treated dorsal skin, draining lymph nodes, liver, and spleen at 1, 6, 24, and 48 h after HA/LNP@MS treatment (Figure S3). Exogenous PGC1α mRNA was reproducibly detected in the treated dorsal skin, showing a time‐dependent decline. In contrast, no reproducible specific amplification was observed in plasma, draining lymph nodes, liver, or spleen at any examined time point (Figure S3A). LC–MS/MS analysis showed that SRT1720 was predominantly retained in the treated dorsal skin, while its levels in plasma, draining lymph nodes, liver, and spleen remained below the lower limit of quantification at all examined time points (Figure S3B). These results indicate that the threaded microneedle system enabled localized delivery of both therapeutic modules with no quantifiable systemic exposure.

### 2.6 Evaluation of SRT1720@HA in Alleviating Fibroblast Senescence

To investigate the effect of SRT1720‐loaded hydrogel (SRT1720@HA), which constitutes the outer layer of the microneedle system, on alleviating fibroblast senescence, we established a senescent model by exposing L929 cells to hydrogen peroxide (Mock) and subsequently treated them with 100% (v/v) hydrogel extract either containing or lacking SRT1720.

**FIGURE 5:**
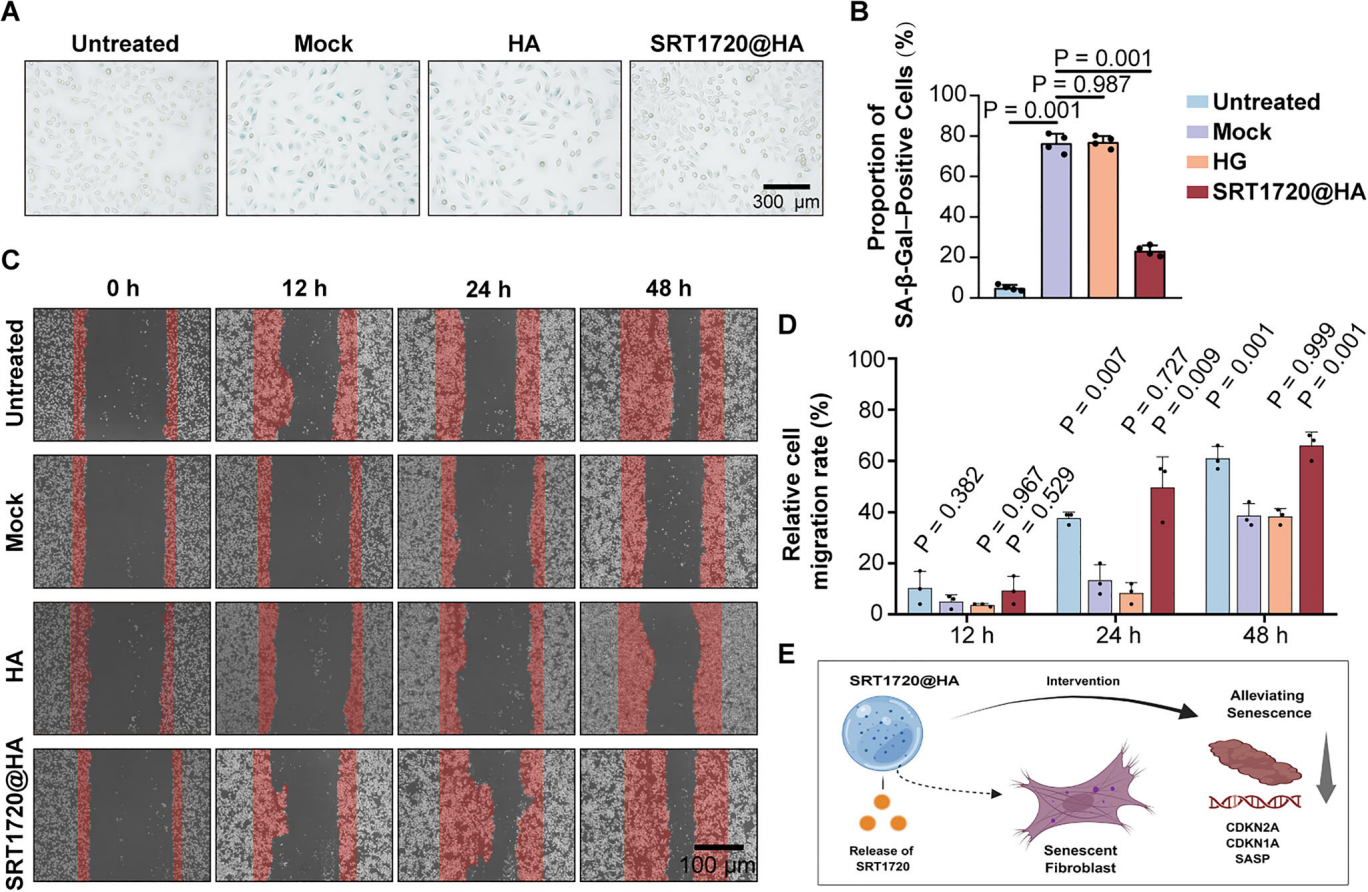
Effects of SRT1720@HA on fibroblast senescence and migratory function. (A) SA‐β‐gal staining of senescent fibroblasts (Mock) treated with HA or SRT1720@HA extract (100% v/v). (B) Quantification plots corresponding to the SA‐β‐gal staining shown in panel A. (C) Scratch assay images of senescent fibroblasts treated with HA or SRT1720@HA extract at 0, 12, 24, and 48 h. (D) Quantification plots corresponding to the scratch closure shown in panel C. (E) Schematic illustration of the proposed mechanism by which SRT1720@HA attenuates fibroblast senescence. All scale bars are shown in the respective panels unless otherwise specified. Statistical analyses were performed using one‐way analysis of variance (ANOVA) followed by Dunnett's multiple‐comparisons test.

**FIGURE 6:**
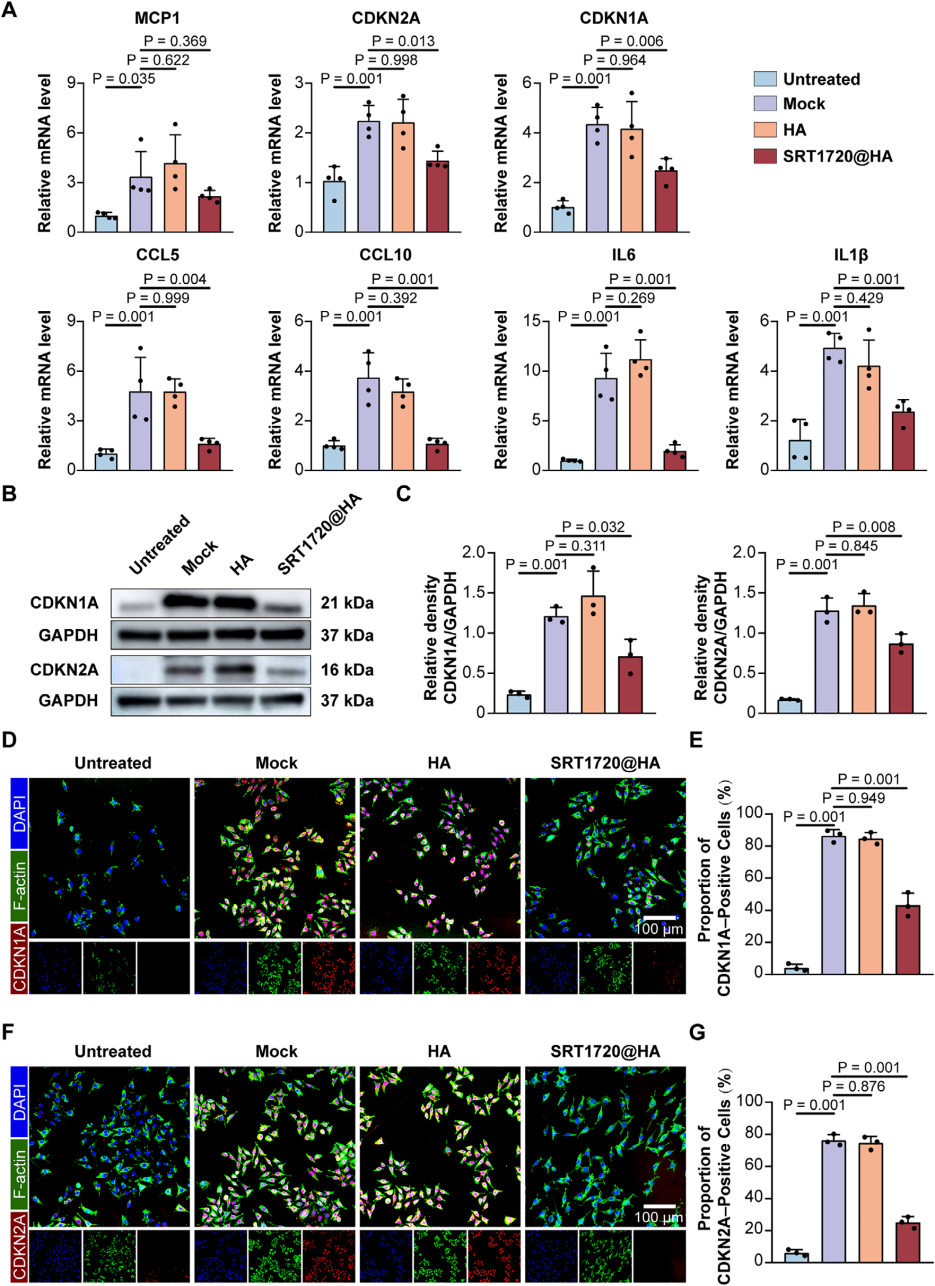
Modulation of senescence‐associated markers in senescent fibroblasts following SRT1720@HA treatment. (A) RT‐qPCR analysis of senescence‐ and inflammation‐related genes in senescent fibroblasts (Mock) treated with HA or SRT1720@HA extract. (B) Western blot analysis of CDKN2A and CDKN1A expression in senescent fibroblasts treated with HA or SRT1720@HA extract. (C) Quantification plots corresponding to the western blot bands shown in panel B. (D) Immunofluorescence staining of CDKN2A and F‐actin in senescent fibroblasts treated with HA or SRT1720@HA extract. Nuclei are counterstained with DAPI. (E) Quantification plots corresponding to the immunofluorescence signals shown in panel D. (F) Immunofluorescence staining of CDKN1A and F‐actin in senescent fibroblasts treated with HA or SRT1720@HA extract. Nuclei are counterstained with DAPI. (G) Quantification plots corresponding to the immunofluorescence signals shown in panel F. All scale bars are shown in the respective panels unless otherwise specified. Statistical analyses were performed using one‐way analysis of variance (ANOVA) followed by Dunnett's multiple‐comparisons test.

β‐galactosidase is a classical marker of cellular senescence, and its positivity rate directly reflects the extent of senescence [42]. Staining results revealed that the proportion of β‐galactosidase–positive cells was significantly reduced in the SRT1720@HA group compared with the Mock group (Figure 5A,B). The migratory capacity of fibroblasts is a key indicator of their functional state, which is typically impaired under senescent conditions [43]. Scratch assay results demonstrated that at 24 h, cells treated with SRT1720@HA exhibited significantly enhanced migration compared with the Mock group (Figure 5C,D). MCP1, CCL5, and CXCL10 are typical inflammatory cytokines frequently upregulated in senescent cells and constitute important components of the senescence‐associated secretory phenotype (SASP). CDKN2A and CDKN1A are canonical cell cycle inhibitors widely recognized as senescence markers, while IL6 and IL1β are core SASP factors closely associated with the pro‐inflammatory microenvironment [44, 45]. RT‐qPCR analysis showed that the expression of these genes were markedly increased in senescent L929 fibroblasts, whereas SRT1720@HA treatment substantially attenuated their upregulation (Figure 6A). Consistent with the transcriptional results, ELISA analysis of the conditioned media further demonstrated reduced secretion of IL‐6 and IL‐1β following SRT1720@HA treatment (Figure S4). Consistently, western blot analysis confirmed that SRT1720@HA markedly reduced the elevated expression of CDKN2A and CDKN1A in senescent fibroblasts, further validating its role in attenuating senescence (Figure 6B,C). Immunofluorescence staining likewise revealed diminished signals of CDKN2A and CDKN1A in the SRT1720@HA group (Figure 6D–G), providing direct spatial evidence that SRT1720@HA suppresses senescence‐associated protein expression. Taken together, these results demonstrate that SRT1720@HA improves multiple hallmarks of fibroblast senescence at phenotypic, functional, and molecular levels, indicating its potential to partially reverse the senescent state and promote functional recovery.

### 2.7 Evaluation of PGC1α@LNP in Attenuating Adipocyte‐to‐Myofibroblast Transition

We next examined the effect of PGC1α‐loaded lipid nanoparticles (PGC1α@LNP), the functional component designed for subcutaneous delivery in the threaded microneedle system, on adipocyte‐to‐myofibroblast transition (AMT). Dermal fibroblast‐to‐myofibroblast transition is a central mechanism of skin fibrosis; accordingly, we first targeted pathological fibroblast activation by reversing the senescence phenotype of dermal fibroblasts with the SRT1720@HA module, as described above. In parallel, our spatial transcriptomic analysis indicated AMT‐related remodeling in the deep dermal adipose compartment and subcutaneous adipose layer. Senescent fibroblasts and their SASP may promote pathological adipose remodeling and AMT, which may in turn further reinforce the local profibrotic microenvironment, forming a reciprocal pathological interaction at the dermal–adipose interface. This finding is consistent with previous studies identifying adiponectin‐positive adipocytic progenitors and dermal adipocytes as direct contributors to myofibroblast formation and cutaneous fibrotic remodeling [38, 46, 47]. Accordingly, differentiated 3T3‐L1 adipocytes were stimulated with TGF‐β1 to establish an AMT model (Mock), followed by treatment with PGC1α@LNP (100 µg/mL total lipid) to evaluate the therapeutic effect of the subcutaneously delivered PGC1α mRNA module.

**FIGURE 7:**
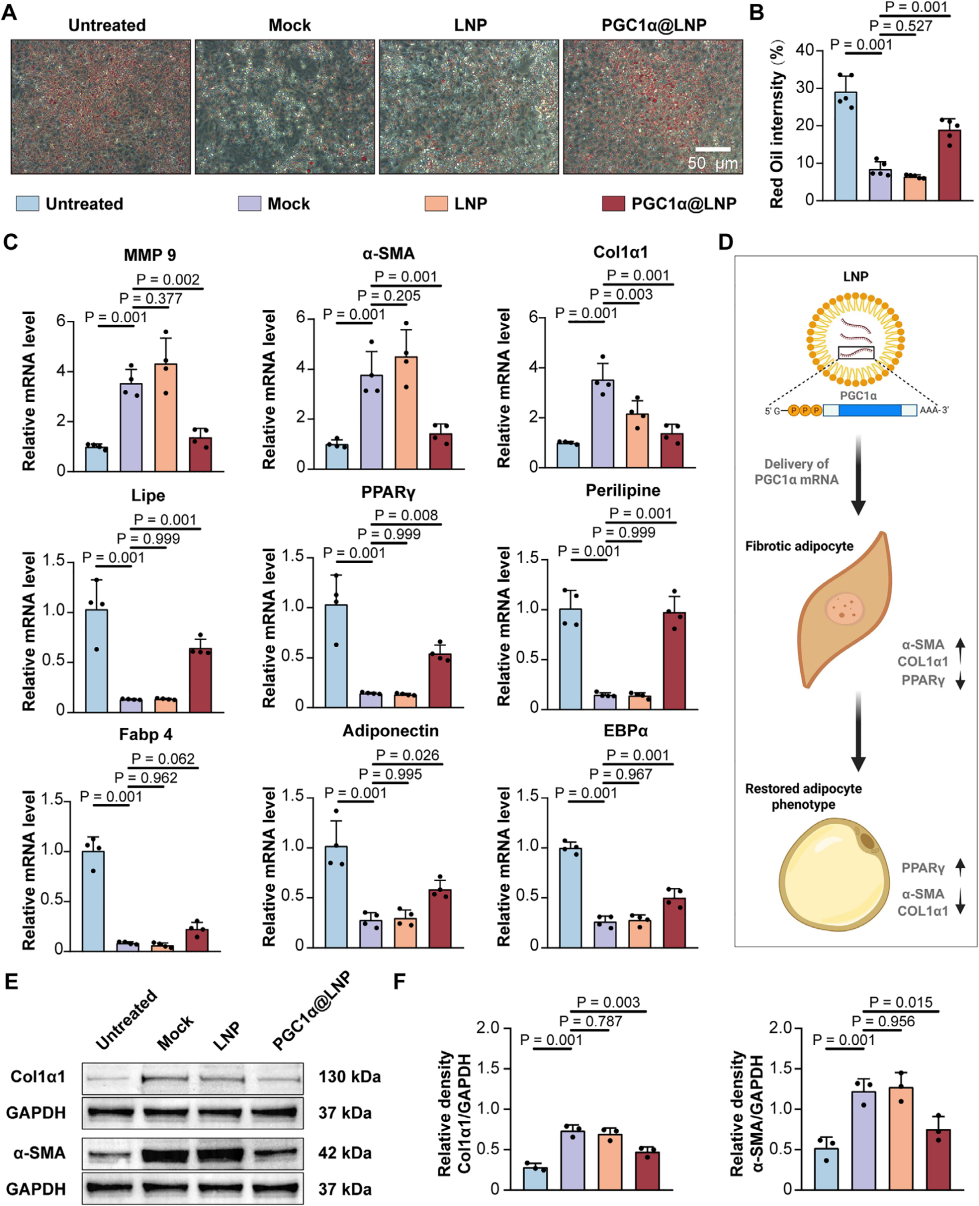
Modulation of adipocyte‐to‐myofibroblast transition in TGF‐β1‐induced 3T3‐L1 adipocytes following PGC1α@LNP treatment. Differentiated 3T3‐L1 adipocytes without TGF‐β1 stimulation served as the Untreated group. TGF‐β1‐stimulated 3T3‐L1 adipocytes were used as the AMT model group (Mock). AMT‐model cells treated with empty LNPs or PGC1α@LNPs were defined as the Empty LNP and PGC1α@LNP groups, respectively. (A) Oil Red O staining of 3T3‐L1 adipocytes in the Untreated, Mock, Empty LNP, and PGC1α@LNP groups. (B) Quantification plots corresponding to the Oil Red O staining shown in panel A. (C) RT‐qPCR analysis of fibrosis‐related and adipocyte‐associated genes in the indicated groups. (D) Schematic illustration of the proposed mechanism by which PGC1α@LNP regulates adipocyte‐to‐myofibroblast transition. (E) Western blot analysis of α‐SMA and Col1a1 expression in the indicated groups. (F) Quantification plots corresponding to the western blot bands shown in panel E. All scale bars are shown in the respective panels unless otherwise specified. Statistical analyses were performed using one‐way analysis of variance (ANOVA) followed by Dunnett's multiple‐comparisons test.

Oil Red O staining, which reflects lipid accumulation and adipogenic differentiation [48], revealed that PGC1α@LNP–treated cells retained markedly more lipid droplets compared with the Mock group, suggesting attenuation of TGFβ1‐induced adipocyte loss and preservation of adipocyte identity (Figure 7A,B). RT‐qPCR analysis further demonstrated that fibrosis‐related markers were significantly downregulated after PGC1α@LNP treatment (Figure 7C). Among them, MMPs involved in extracellular matrix remodeling, α‐SMA as a hallmark of myofibroblast activation, and Col1a1 reflecting collagen deposition all showed marked reductions [49, 50]. In contrast, adipocyte‐associated genes were upregulated, including the lipid metabolism marker Lipe, the adipogenic transcription factors PPARγ and C/EBPα, as well as Perilipin, Fabp4, and Adiponectin, which are indicative of adipocyte differentiation and functional maintenance [51, 52]. These results indicate that PGC1α@LNP effectively suppresses fibrotic reprogramming while restoring adipocyte characteristics. At the protein level, western blotting further demonstrated that PGC1α@LNP intervention reduced the elevated protein levels of α‐SMA and Col1a1 in the AMT model, confirming its inhibitory effect on myofibroblastic transition (Figure 7E,F).

Altogether, these findings suggest that PGC1α@LNP effectively suppresses TGFβ1‐induced adipocyte‐to‐myofibroblast transition at both phenotypic and molecular levels, thereby maintaining adipocyte characteristics and attenuating fibrotic reprogramming.

### 2.8 Therapeutic Efficacy of the Threaded Microneedle System in a Bleomycin‐induced Skin Fibrosis Model

**FIGURE 8:**
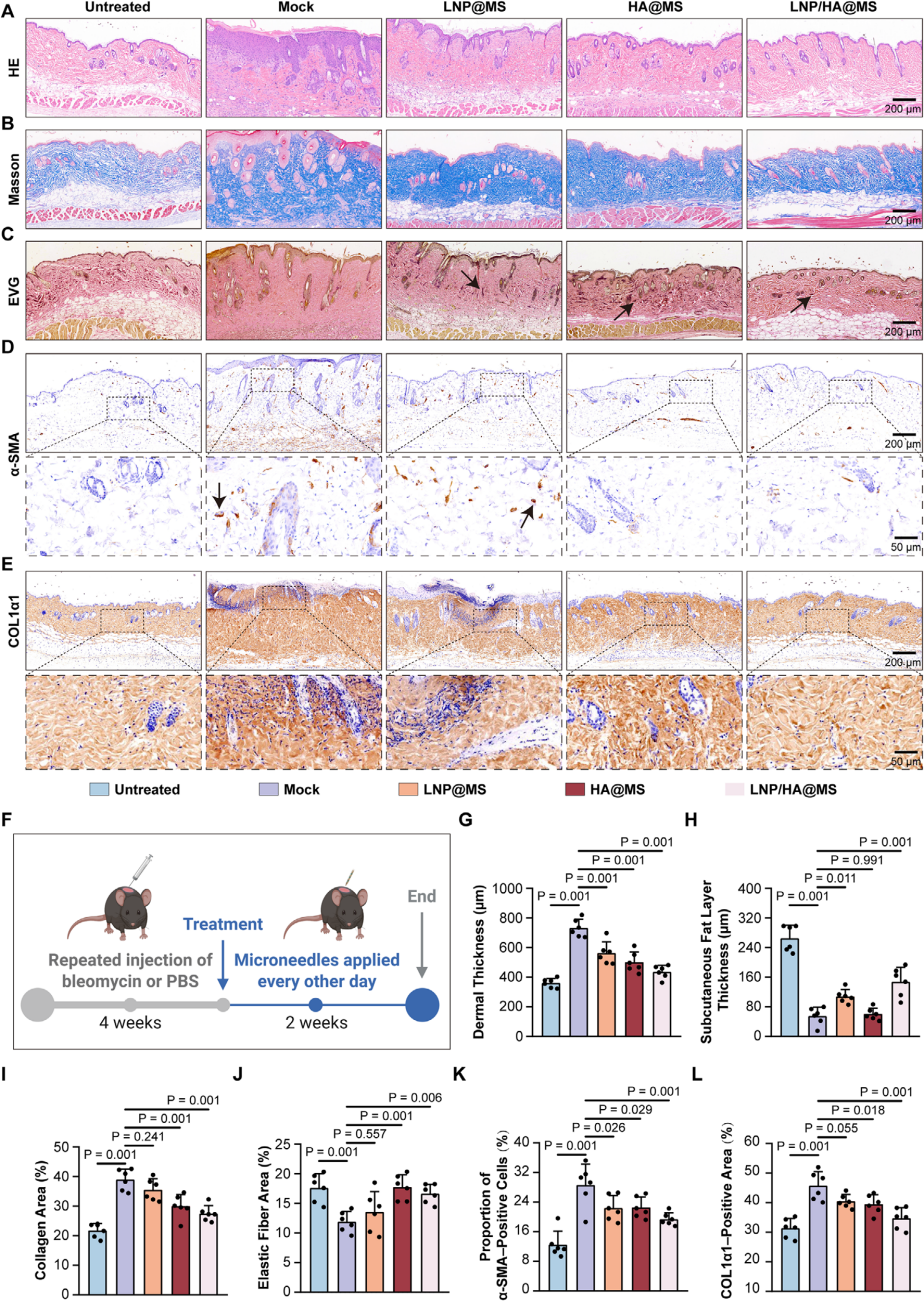
In vivo antifibrotic effects of threaded microneedle–mediated delivery in a bleomycin‐induced skin fibrosis mouse model. Groups: Normal, healthy mice without bleomycin or microneedle treatment; Mock, fibrotic mice treated with uncoated microneedles; LNP@MS, fibrotic mice treated with PGC1α@LNP‐loaded microneedles; HA@MS, fibrotic mice treated with SRT1720@HA‐coated microneedles; and HA/LNP@MS, fibrotic mice treated with dual‐loaded microneedles. (A) H&E staining of dorsal skin. (B) Masson's trichrome staining of dorsal skin, in which collagen fibers were stained blue, cytoplasm and muscle fibers were stained red. (C) EVG staining of dorsal skin showing elastic fibers stained black; representative elastic fibers are indicated by black arrows. (D) Immunohistochemical staining of α‐SMA, with positively stained myofibroblasts appearing brown and indicated by black arrows. (E) Immunohistochemical staining of COL1A1, with COL1A1‐positive areas appearing brown. (F) Schematic illustration of the bleomycin‐induced skin fibrosis model and microneedle‐based intervention workflow. (G) Quantification of dermal thickness corresponding to panel A. (H) Quantification of subcutaneous fat layer thickness corresponding to panel A. (I) Quantification of collagen‐positive area corresponding to panel B. (J) Quantification of elastic fiber area corresponding to panel C. (K) Quantification of α‐SMA‐positive cells corresponding to panel D. (L) Quantification of the COL1A1‐positive area corresponding to panel E. Scale bars are indicated in the corresponding panels. Statistical analyses were performed using one‐way analysis of variance (ANOVA), followed by Dunnett's multiple‐comparisons test.

The ultimate goal of our study was to achieve precise drug delivery across different skin layers using the threaded microneedle system, thereby simultaneously targeting key pathological processes in both the dermis and subcutaneous fat to alleviate fibrotic remodeling. To this end, we performed in vivo validation in a bleomycin‐induced skin fibrosis model. After model establishment, animals were randomly allocated to the indicated groups. The untreated Scleroderma group received no microneedle intervention, whereas the Mock group received uncoated microneedle insertion following the same schedule and procedure used for the treatment groups. The Mock group was included to control for the potential effects of mechanical stimulation, as needling itself may influence the local tissue microenvironment by inducing sterile inflammation, deforming connective tissue, and activating mechanosensitive cells [53]. As shown in Figure S5, the untreated Scleroderma group exhibited pronounced dermal thickening, increased collagen deposition, and reduced subcutaneous adipose tissue compared with the Normal group, confirming successful model establishment. No significant differences were observed between the untreated Scleroderma and Mock groups, indicating that uncoated microneedle insertion alone did not substantially alter the fibrotic phenotype. The treatment groups received microneedle‐based delivery of the outer SRT1720@HA hydrogel coating (HA@MS), groove‐loaded PGC1α@LNP (LNP@MS), or the combined formulation (HA/LNP@MS) (Figure 8F). During the treatment phase, microneedles were applied every other day to the fibrotic skin, with evenly distributed punctures to ensure consistent local intervention.

The threaded microneedle system achieves differential spatial delivery through the distinct loading configurations of its two therapeutic modules. The spiral grooves along the distal drug‐loading region provide recessed spaces that protect the lyophilized PGC1α@LNP formulation from premature displacement during insertion and facilitate its transport to deeper tissue layers [41]. Following insertion and rotational manipulation, tissue hydration and mechanical shear promote the release of the groove‐confined LNP formulation along the insertion tract, including both the dermal and subcutaneous regions [54]. This interpretation is supported by fluorescence tracing in the simulated skin model and murine skin, which demonstrated deeper, tract‐wide distribution of PGC1α@LNP (Figure 3C,D). In contrast, SRT1720@HA was applied as an external hydrogel coating. During insertion, tissue resistance promoted preferential removal and retention of this surface coating within the dermis, representing a delivery behavior similar to that of conventional coated needles. After deposition, the HA matrix absorbed interstitial fluid, underwent swelling, and released SRT1720 within the dermal compartment [41]. This distribution enables SRT1720 to target senescent dermal fibroblasts and suppress their profibrotic secretory phenotype [55]. Meanwhile, the PGC1α@LNP formulation distributed along the deeper insertion tract protected the encapsulated mRNA, supported sustained release, and enabled intracellular delivery and translation in adipocytes [28]. Delivery of this module to the deep dermal and subcutaneous adipose compartments was intended to restore adipocyte metabolic and differentiation homeostasis and suppress adipocyte‐to‐myofibroblast transition [56]. Thus, the complementary distribution of the two modules provides a structural basis for simultaneously targeting dermal fibroblast senescence and pathological adipose remodeling. Nevertheless, the absence of dose‐matched free SRT1720 and PGC1α@LNP controls limits our ability to distinguish the effects of the therapeutic cargos from those of the threaded microneedle platform, which warrants further investigation.

#### 2.8.1 In Vivo Antifibrotic Efficacy of the Threaded Microneedle System in a Bleomycin‐induced Skin Fibrosis Model

After two weeks of regular intervention, histological and molecular analyses were performed on the fibrotic dorsal skin. In the Mock group, skin tissues exhibited pronounced dermal thickening with disorganized collagen bundles and marked thinning of the subcutaneous fat layer (Figure 8A). Masson and EVG staining further revealed extensive and dense collagen accumulation along with fragmented and sparse elastic fibers, leading to near‐complete loss of the elastic fiber network, indicating severe fibrotic remodeling (Figure 8B,C). In contrast, all three microneedle‐based treatments mitigated fibrotic manifestations, with HA/LNP@MS showing the most pronounced improvement: dermal thickness was markedly reduced, collagen deposition significantly attenuated, and elastic fiber architecture substantially restored (Figure 8G–J). Layer‐specific effects were also evident: HA@MS primarily acted on the dermis, reducing collagen accumulation and promoting elastic fiber remodeling, while LNP@MS was more effective in restoring subcutaneous fat thickness but showed limited effects on elastic fibers when used alone. Immunohistochemical analysis supported these observations, showing strong upregulation of COL1A1 and α‐SMA in the Mock group, whereas all interventions significantly reduced their expression, with the greatest suppression observed in the HA/LNP@MS group (Figure 8D,E,K,L). However, because the animals were evaluated only at the end of the two‐week treatment period, the durability of the therapeutic effects and the possibility of fibrosis recurrence after treatment cessation remain to be determined in future longitudinal studies.

#### 2.8.2 Layer‐Specific Modulation of Dermal Senescence and Adipocyte–Myofibroblast Transition

**FIGURE 9:**
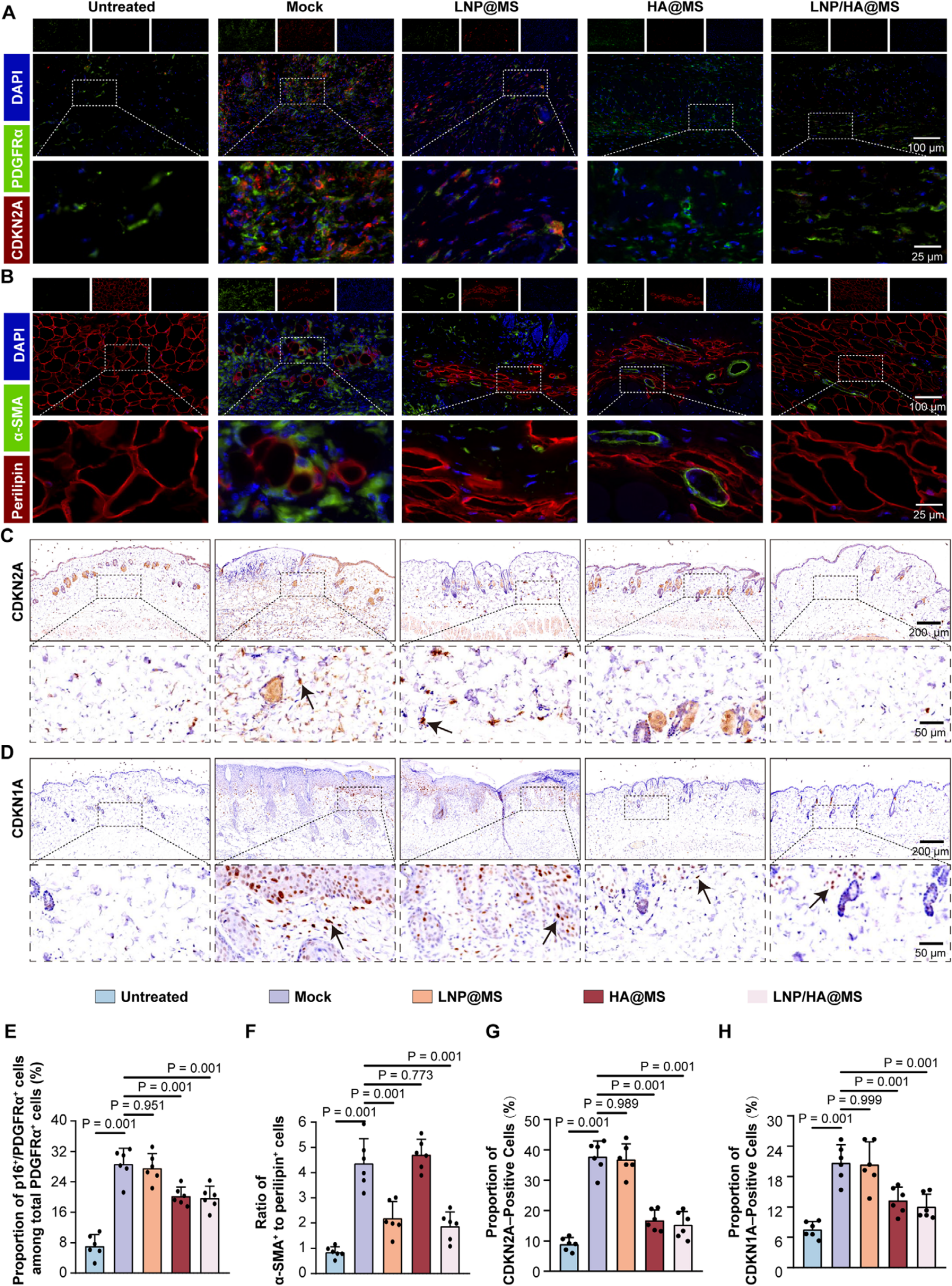
Layer‐specific assessment of dermal senescence and adipocyte‐to‐myofibroblast transition following microneedle‐based treatment. (A) Double immunofluorescence staining of CDKN2A (red), PDGFRα (green), and DAPI (blue) in dorsal skin. (B) Double immunofluorescence staining of α‐SMA (green), Perilipin (red), and DAPI (blue) in the subcutaneous layer. (C) Immunohistochemical staining of CDKN2A in dorsal skin, with positive staining shown in brown and indicated by black arrows. (D) Immunohistochemical staining of CDKN1A in dorsal skin, with positive staining shown in brown and indicated by black arrows. (E) Quantification of CDKN2A^+^/PDGFRα^+^ double‐positive cells expressed as a ratio to total PDGFRα^+^ cells, corresponding to panel A. (F) Quantification of α‐SMA^+^/Perilipin^+^ ratio corresponding to panel B. (G) Quantification of CDKN2A‐positive cell proportion corresponding to panel C. (H) Quantification of CDKN1A‐positive cell proportion corresponding to panel D. All scale bars are shown in the respective panels unless otherwise specified. Statistical analyses were performed using one‐way analysis of variance (ANOVA) followed by Dunnett's multiple‐comparisons test.

To further explore the layered mechanisms of the threaded microneedle system, we assessed dermal senescence and AMT. Double immunofluorescence for PDGFRα and p16 revealed abundant accumulation of p16^+^/PDGFRα^+^ fibroblasts in the Mock group, indicating enrichment of senescent fibroblasts (Figure 9A). Both HA@MS and HA/LNP@MS markedly reduced the proportion of senescent fibroblasts, whereas LNP@MS showed no significant effect (Figure 9E). This trend was consistent with immunohistochemical staining for p16 and p21, further confirming the role of the hydrogel module in alleviating dermal senescence (Figure 9C,D,G,H). Meanwhile, α‐SMA and Perilipin co‐staining highlighted active AMT in the Mock group, evidenced by increased α‐SMA^+^ cells and reduced Perilipin^+^ adipocytes within the subcutaneous layer (Figure 9B). Both LNP@MS and HA/LNP@MS significantly reduced the α‐SMA^+^/Perilipin^+^ ratio, indicating effective suppression of AMT, while HA@MS showed no apparent effect (Figure 9F).

### 2.9 Transcriptomic Reprogramming Underlying Cross‐Layer Therapeutic Synergy

**FIGURE 10:**
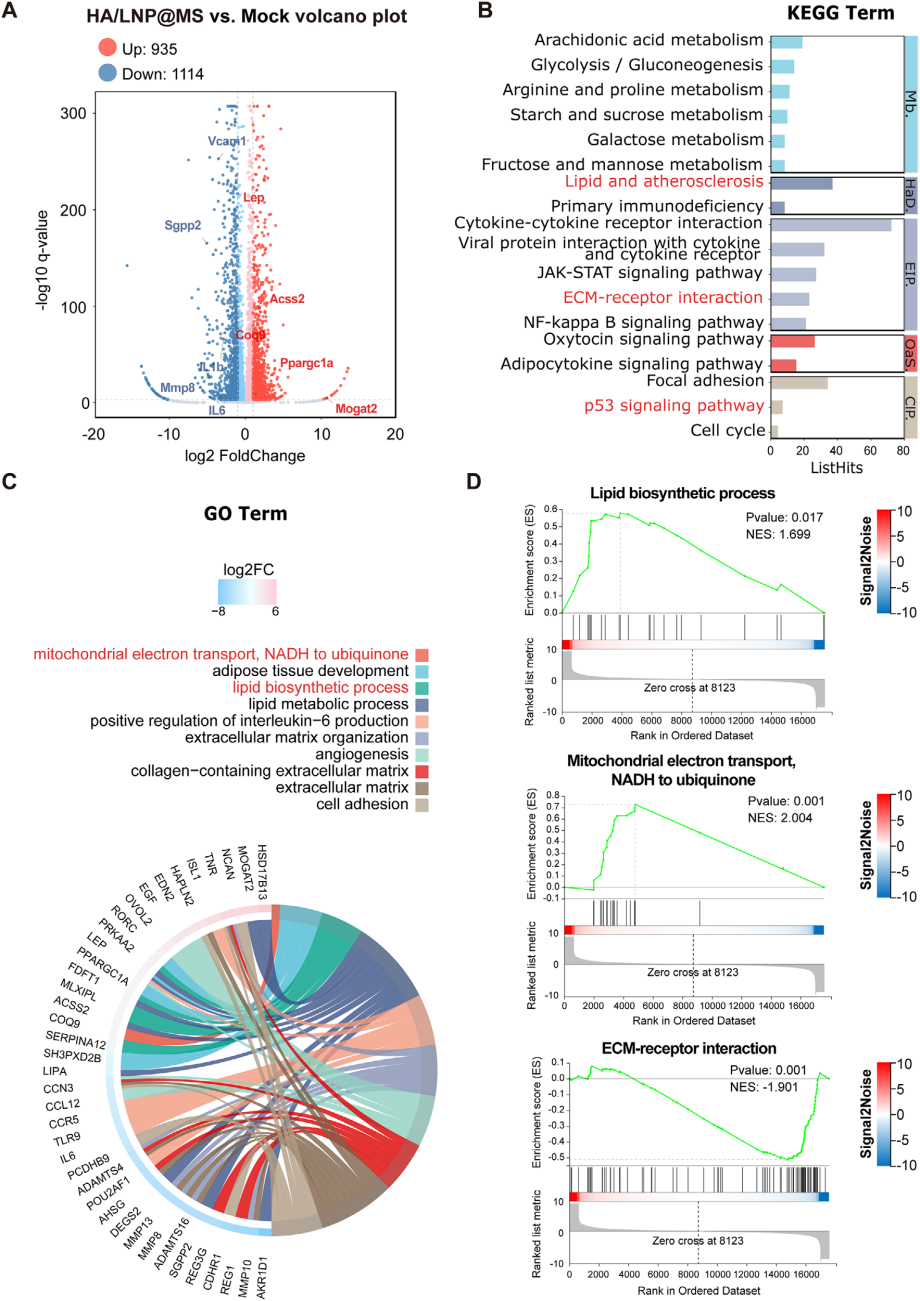
Transcriptomic profiling of fibrotic skin following HA/LNP@MS treatment. Groups: Mock (bleomycin‐induced skin fibrosis) and HA/LNP@MS. (A) Volcano plot showing differentially expressed genes between the groups. (B) KEGG pathway enrichment analysis of differentially expressed genes. (C) GO enrichment analysis of differentially expressed genes. (D) GSEA analysis of selected pathways comparing the Mock and HA/LNP@MS groups.

To further elucidate the molecular mechanisms underlying the therapeutic effects, transcriptomic sequencing was conducted on fibrotic skin from the Mock and HA/LNP@MS groups (Figure 10). Differential expression analysis revealed extensive transcriptional reprogramming after HA/LNP@MS treatment, marked by the downregulation of senescence‐ and inflammation‐associated pathways such as p53 signaling pathway. Key inflammatory and matrix‐remodeling genes, including Il1b, IL6, Mmp8, Vcam1, and Sgpp2 were significantly suppressed, consistent with the alleviation of chronic inflammation and extracellular matrix overproduction [57]. Correspondingly, pathways involved in ECM–receptor interaction and fibrotic remodeling were prominently attenuated, aligning with the histological reduction of collagen deposition [33]. Conversely, HA/LNP@MS treatment reactivated adipogenic and metabolic programs, reflected by the upregulation of Lep, Acss2, Coq9, Ppargc1a, and Mogat2, genes essential for lipid biosynthesis, mitochondrial electron transport, and PPAR‐dependent metabolic regulation [58, 59]. RT‐qPCR validation further confirmed that Il1β and Il6 were downregulated, whereas Ppargc1α and Lep were upregulated following HA/LNP@MS treatment, consistent with the RNA‐seq findings (Figure S6). Enrichment analysis further confirmed enhanced lipid biosynthetic and mitochondrial electron transport processes, suggesting restoration of mitochondrial function and adipocyte lineage stability within the subcutaneous layer. Collectively, these transcriptomic signatures indicate that the modular LNP–hydrogel integrated threaded microneedles concurrently suppress pro‐fibrotic, inflammatory, and senescence‐associated cascades while restoring lipid metabolism and energy homeostasis, thereby supporting its layered therapeutic effects across dermal and subcutaneous tissues.

Overall, these results demonstrate that the threaded microneedle system exerts stratified therapeutic effects in vivo: the hydrogel module alleviates dermal fibrosis primarily by attenuating fibroblast senescence, while the lipid nanoparticle module restores subcutaneous fat homeostasis by inhibiting AMT. When combined, the dual‐module system achieves synergistic cross‐layer regulation, effectively disrupting pathological feedback loops and ultimately producing a more comprehensive antifibrotic outcome in fibrotic skin.

## 3 Conclusion

In this study, we developed a threaded microneedle system with a simple modular design. It combines a hydrogel coating and lipid nanoparticles to deliver drugs to different skin layers. SRT1720@HA is coated on the needle surface and releases quickly after insertion. PGC1α@LNP is loaded in the helical grooves and releases more slowly. In this way, the system delivers drugs to the dermis and subcutaneous fat at the same time with one treatment. Material tests and biocompatibility assays showed that both parts are suitable for skin use. SRT1720@HA reduced senescence in dermal fibroblasts. PGC1α@LNP inhibited adipocyte‐to‐myofibroblast transition and improved adipocyte metabolic function. In animal experiments, the threaded microneedles improved collagen structure in the dermis and reduced subcutaneous fat loss. The treatment repaired both skin layers in a coordinated way. Transcriptome analysis showed that the combined treatment reduced genes related to senescence, inflammation, and fibrosis. At the same time, it increased genes related to lipid metabolism and mitochondrial function. These results explain how the layered delivery works at the molecular level. Together, these findings demonstrate the therapeutic potential of the threaded microneedle system in the mouse model. Although this model does not fully recapitulate human skin fibrosis, our findings highlight the potential of this system as a minimally invasive strategy for skin diseases involving deep and multilayer tissue remodeling. Clinical validation will be required to further assess its translational applicability, and future work will focus on optimizing drug release profiles and device design to support clinical application.

## 4 Methods

### 4.1 Preparation and Characterization of PGC1α@LNP

Mouse PGC1α mRNA was synthesized by Unigen Biotech Co. (Suzhou, China), with its nucleotide sequence provided in Table S2. According to the manufacturer's quality control report, the mRNA purity was ≥90%, the A260/A280 ratio was 1.9–2.2, and the concentration was 1.0 mg/mL (±3%). Lipid nanoparticles (LNPs) were prepared using a microfluidic mixing method [60, 61]. Briefly, ionizable lipids, cholesterol, DSPC, and PEG‐lipid were dissolved in absolute ethanol at a molar ratio of 50:38.5:10:1.5. PGC1α mRNA solution [62] was prepared in 25 mM sodium acetate buffer (pH 4.0). The organic and aqueous phases were mixed at a flow rate ratio of 1:3 through a microfluidic chip to form LNPs encapsulating PGC1α (PGC1α@LNP). The resulting suspension was dialyzed against PBS (pH 7.4) for 2 h to remove ethanol and unincorporated materials. For fluorescence tracing, DiO was incorporated into the lipid phase.

The particle size, polydispersity index (PDI), and zeta potential were measured using dynamic light scattering (DLS, Zetasizer Nano ZS90, Malvern Instruments, UK). The morphology was observed using transmission electron microscopy (TEM, JEOL JEM‐2100) after negative staining with 2% phosphotungstic acid. Encapsulation efficiency and mRNA loading were determined using the Quant‐iT RiboGreen RNA assay. Unencapsulated and total mRNA were measured before and after LNP disruption with 1% Triton X‐100, respectively, and calculated as follows:

$$\begin{array}{ccc} EE\;\left(\%\right) & = & \frac{\left(C_{total}-C_{free}\right)}{C_{total}}\;\times 100\%,\;\;\\ mRNA\;loading\left(\mu g/\mathrm{mg}\right) & = & \frac{m_{encapsulated\;mRNA}}{m_{total\;lipid}} \end{array}$$

where C*_total_* and C*_free_* represent the total and unencapsulated mRNA concentrations respectively, and *m*_*encapsulated* *mRNA*_ = (*C_total_* − *C_free_*)  × *V*.

For storage and microneedle loading, PGC1α@LNP was frozen at −80°C for 2 h, lyophilized overnight, and stored at −20°C under sealed, desiccated conditions for up to one month. To evaluate functional stability, freshly prepared PGC1α@LNP, formulations reconstituted immediately after lyophilization, and formulations reconstituted after one month of storage were compared. Equivalent mRNA doses from each formulation were delivered to 3T3‐L1 cells, followed by assessment of PGC1α mRNA and protein expression using RT‐qPCR and Western blotting, respectively.

### 4.2 Preparation, Rheological Analysis, and Drug Release of SRT1720@HA Hydrogel

SRT1720 was dissolved in DMSO and mixed with a 2% (w/v) sodium hyaluronate solution under gentle stirring to form a homogeneous SRT1720@HA hydrogel with a final SRT1720 concentration of 3 mM [63]. For fluorescence tracing experiments, Rhodamine B was incorporated into the hydrogel to visualize the hydrogel coating.

The viscoelastic properties were measured using a rotational rheometer (MCR 302, Anton Paar, Austria) with a 25 mm parallel plate at 25°C. The storage modulus (G′) and loss modulus (G″) were recorded as a function of oscillatory strain from 0.1% to 100%. For in vitro release, 1 mL of SRT1720@HA was placed in a dialysis bag (MWCO 8–14 kDa) and incubated in 20 mL PBS (pH 7.4, 37°C) under shaking at 100 rpm. At predetermined time points, 1 mL of the medium was collected and replaced with fresh PBS. The SRT1720 concentration in the hydrogel extracts and release medium was determined by UV‐vis spectrophotometry at 320 nm and used to calculate the actual drug loading (mg/mL), loading efficiency (%), and cumulative release (%).

### 4.3 Preparation and Layered Coating of Threaded Microneedles

Single‐threaded microneedles were used in this study. As previously reported, threaded microneedles have been successfully applied for lesion‐localized drug delivery in osteoarthritic rats [29, 32]. The threaded microneedles were fabricated by introducing spiral grooves with defined dimensions onto stainless‐steel needles, and the groove structure could be customized according to the target experimental model [31]. To adapt the microneedles for cutaneous delivery in C57BL/6 mice, each threaded microneedle used in the present study had a total length of 4 mm and an outer diameter of 230 µm. The distal 2 mm of the microneedle contained the threaded groove structure, whereas the proximal 2 mm was non‐threaded. Drug loading was restricted to the distal 1.5 mm of the threaded region.

For layered loading, a thin layer of 2% (w/v) sodium hyaluronate solution was first applied to the spiral grooves to facilitate LNP deposition. Because the amount retained by an individual microneedle was below the reliable weighing range of high‐capacity microbalance (Cubis II, Sartorius, Germany), PGC1α@LNP loading was performed and quantified in batches. A total of 0.3 mg of lyophilized PGC1α@LNP was applied to a batch of 100 microneedles. After loading, the unbound formulation was collected and quantified, and the average loading per microneedle was calculated from the total retained amount divided by the number of microneedles. The resulting microneedles were lyophilized to stabilize the groove‐confined LNP layer and stored at −20°C. Prior to application, the microneedles were immersed for 1 min in Rhodamine B‐containing SRT1720@HA hydrogel, allowing the drug‐loaded hydrogel to form an outer coating over the threaded drug‐loading region. The amount of SRT1720 retained on microneedles was quantified by LC–MS/MS (SCIEX QTRAP 4500) following extraction from the coated microneedles. This procedure generated a modular coating structure consisting of groove‐embedded PGC1α@LNPs and an outer SRT1720@HA hydrogel layer. The coating uniformity was observed using optical microscopy (Leica DVM6) and fluorescence microscopy (Zeiss LSM880) [64].

### 4.4 Clinical Sample Collection and Preparation

Full‐thickness skin samples were taken from fibrotic lesions of patients with localized scleroderma. Skin samples from healthy controls were taken from the same body sites during routine surgical procedures. All procedures followed protocols approved by the institutional ethics committee (Ethics Approval No. SH9H‐2020‐T138‐2). All participants signed written informed consent before sample collection. After removal, the samples were washed with cold phosphate‐buffered saline (PBS) to remove blood. Then the tissues were fixed in 4% paraformaldehyde, embedded in paraffin, and cut into 5 µm sections.

### 4.5 Biocompatibility Assessment

L929 and 3T3‐L1 cells were used to evaluate the cytocompatibility of SRT1720@HA and PGC1α@LNP, respectively. Cells were cultured in high‐glucose Dulbecco's Modified Eagle Medium (DMEM; Gibco, USA) supplemented with 10% fetal bovine serum (FBS; Gibco, USA) under standard conditions (37°C, 5% CO_2_). Cytotoxicity was assessed using a CCK‐8 assay (Cell Counting Kit‐8; Cat. No. C0038, Beyotime, Shanghai, China). Cells were seeded into 96‐well plates (1 × 10^4^ cells/well), exposed to hydrogel extracts or nanoliposomes, and then incubated with 10% (v/v) CCK‐8 reagent for 1 h at 37°C. Absorbance was recorded at 450 nm using a microplate reader (SpectraMax iD3, Molecular Devices, USA).

Cell proliferation was further examined using an EdU Cell Proliferation Kit (Cat. No. C0071S, Beyotime, China). After 24 h treatment, cells were incubated with EdU solution (50 µM, 2 h), fixed, permeabilized, and counterstained with Hoechst 33342. EdU‐positive nuclei were visualized under an inverted fluorescence microscope (EVOS M5000, Thermo Fisher Scientific, USA), and quantified with ImageJ software. Cell viability was also evaluated by Calcein‐AM/propidium iodide (PI) double staining (Live/Dead Cell Viability Kit; Cat. No. C2015S, Beyotime, China). After 30 min incubation at 37°C, live and dead cells were distinguished by green (Calcein‐AM) and red (PI) fluorescence, respectively, and analyzed by fluorescence microscopy with ImageJ quantification.

### 4.6 Systemic Exposure Assessment

To evaluate systemic exposure after local microneedle application, plasma, treated dorsal skin, draining lymph nodes, liver, and spleen were collected at 1, 6, 24, and 48 h after HA/LNP@MS treatment. Blood samples were collected and centrifuged to obtain plasma. Tissue samples were rinsed with cold PBS, weighed, and homogenized.

Delivered PGC1α mRNA was analyzed by RT‐qPCR using primers specific to the exogenous PGC1α mRNA sequence used in this study. Samples showing reproducible specific amplification across technical replicates were considered detectable. For samples with detectable amplification, relative PGC1α mRNA levels were calculated using the treated dorsal skin collected at 1 h as the calibrator. Samples without reproducible specific amplification were recorded as not detected. SRT1720 levels in plasma and tissue homogenates were quantified by LC–MS/MS (SCIEX QTRAP 4500). Calibration curves were prepared in blank plasma or the corresponding blank tissue homogenates, and the results were expressed as ng/mL plasma or ng/g tissue.

### 4.7 Preparation of Cellular Experimental Models and Interventions

Two cellular models were established to evaluate the layer‐specific therapeutic effects of the two delivery modules. To establish the senescence model, L929 cells were seeded in six‐well plates at 2 × 10^5^ cells per well and treated with 200 µM H_2_O_2_ for 6 h to induce oxidative stress. After treatment, the medium was changed to fresh complete medium. Cells were then cultured under normal conditions to allow senescence‐related changes to appear.

To build the adipocyte‐to‐myofibroblast transition (AMT) model, 3T3‐L1 cells were seeded in 0.1% gelatin‐coated six‐well plates at 2 × 10^5^ cells per well and cultured in complete medium until 80% confluence. The cells were then induced to differentiate into mature adipocytes using OriCell induction medium A for 3 days, followed by induction medium B for 1 day (OriCell, MUXTL‐90031). Medium A and medium B were subsequently alternated until lipid droplets were observed. Differentiated adipocytes were then treated with TGF‐β1 (10 ng/mL) for 24 h to induce AMT.

For intervention studies, senescent L929 cells were incubated for 24 h with 100% (v/v) hydrogel extract prepared from unloaded HA hydrogel or SRT1720‐loaded HA hydrogel (SRT1720@HA; MedChemExpress, Cat. No. HY‐10532). The 3T3‐L1 adipocyte‐derived myofibroblast‐like cells were treated for 24 h with empty LNP or PGC1α@LNP at an equivalent total lipid concentration of 100 µg/mL to evaluate their effects on AMT.

### 4.8 Oil Red O Staining

3T3‐L1 cells were washed with PBS and fixed in 4% paraformaldehyde for 20 min at room temperature. After equilibration with 60% isopropanol for 5 min, the cells were incubated with freshly prepared Oil Red O working solution (OriCell, OILR‐10001) for 30 min. Excess dye was gently rinsed off with distilled water until the background became clear, and images were obtained using an optical microscope [65].

### 4.9 Senescence‐associated β‐Galactosidase (SA‐β‐Gal) Staining

L929 cells were seeded into 6‐well plates and subjected to the indicated treatments. After treatment, cells were rinsed with PBS and fixed with 2% paraformaldehyde for 10 min at room temperature. Following fixation, the cells were washed again with PBS and then incubated with the SA‐β‐Gal staining working solution (Beyotime, China) at 37°C in a CO_2_‐free and light‐protected environment for 2 h. Stained cells were examined using a confocal laser scanning microscope (Leica, Germany), and the fluorescence intensity of SA‐β‐Gal was quantified using ImageJ software.

### 4.10 Wound Healing Assays

L929 cells were seeded into 6‐well plates and subjected to the indicated treatments. A linear scratch was then generated across the cell monolayer using a sterile 200 µL pipette tip, and detached cells were removed by rinsing three times with PBS. The cultures were subsequently maintained under uniform conditions. Wound closure was monitored at 0, 12, 24, and 48 h using an inverted fluorescence microscope (EVOS M5000, Thermo Fisher Scientific, USA). The migration of cells into the wound area was quantified by measuring the scratch width at three randomly selected regions using ImageJ software.

### 4.11 WB Analyses

Cells were lysed in pre‐chilled RIPA buffer (Beyotime, Beijing, China) on ice for 10 min, and the lysates were centrifuged at 12,000 rpm (13 500× *g*) for 10 min at 4°C to remove cellular debris. The resulting supernatants were collected, and protein concentrations were determined using a BCA Protein Assay Kit (Thermo Fisher Scientific, USA). Equal amounts of protein (10 µg per sample) were separated by 10% SDS‐PAGE at 150 V for 40 min and subsequently transferred onto nitrocellulose membranes (Millipore, Bedford, MA, USA). Membranes were blocked with 5% bovine serum albumin (BSA) for 1 h at room temperature and incubated with the following primary antibodies: α‐SMA (Cell Signaling Technology [CST], Cat. No. 19245S), COL1A1 (CST, Cat. No. 72026S), CDKN1A (CST, Cat. No. 2947), CDKN2A (CST, Cat. No. 23200), PGC1α (Abcam, Cat. No. ab191838), and GAPDH (CST, Cat. No. 5174S). After washing, membranes were incubated with horseradish peroxidase (HRP)‐conjugated secondary antibody (anti‐rabbit IgG, CST, Cat. No. 7074S). Protein bands were visualized by chemiluminescence and quantified using ImageJ software.

### 4.12 RT‐qPCR

Total RNA was isolated using TRIzol reagent (Invitrogen, Carlsbad, CA, USA) according to the manufacturer's instructions. Complementary DNA (cDNA) was synthesized from 1 µg of total RNA using a reverse transcription kit (Takara, Japan). Quantitative PCR was performed with SYBR Green Master Mix (Takara, Japan) on a real‐time PCR detection system (ABI 7500, Applied Biosystems, USA). Relative gene expression was calculated using the 2^−ΔΔCt^ method, with GAPDH as the internal control. Primer sequences used in this study are listed in Table S1.

### 4.13 Immunofluorescence Staining (Cell Culture)

Cells grown on sterile glass coverslips were fixed with 4% paraformaldehyde in PBS for 10 min at room temperature and permeabilized with 0.5% Triton X‐100 in PBS for 10 min. After blocking with 1% BSA in PBS for 30 min, cells were rinsed three times with PBS and incubated with primary antibodies for 12 h at 4°C. After washing, cells were incubated with FITC‐ or Alexa Fluor‐conjugated secondary antibodies for 1 h at room temperature. F‐actin was stained with phalloidin (green), and nuclei were counterstained with DAPI (blue). Immunofluorescence images were acquired using a laser scanning confocal microscope (Leica, Germany).

### 4.14 Enzyme‐Linked Immunosorbent Assay (ELISA) of IL‐6 and IL‐1β

Following senescence induction and treatment with the indicated formulations, the cell culture supernatants were collected and centrifuged to remove cellular debris. The concentrations of IL‐6 and IL‐1β in the supernatants were quantified using mouse IL‐6 and mouse IL‐1β ELISA kits (Abcam, Cambridge, UK) according to the manufacturer's instructions. Absorbance was measured at 450 nm using a microplate reader (SpectraMax iD3, Molecular Devices, USA), and cytokine concentrations were calculated from the corresponding standard curves.

### 4.15 Preparation of Animal Experimental Model

All animal procedures were conducted in accordance with institutional guidelines and approved by the Animal Ethics Committee. Male C57BL/6 mice (6 weeks old) were purchased from Shanghai Model Organisms Center (Shanghai, China) and acclimatized for one week before experimentation. To establish the bleomycin‐induced skin fibrosis model, a 1 cm × 1 cm area was marked on the midline dorsal skin of each mouse. Mice received daily intradermal injection of bleomycin solution at the center of the marked area for 28 consecutive days. Bleomycin was prepared at a concentration of 1 mg/mL in PBS, and 100 µL was injected per mouse per day. Control mice received 100 µL PBS at the same site using the same injection procedure.

### 4.16 Grouping and Treatment of Animal Experiments

Before model establishment, mice were randomly assigned to six groups: (i) Normal group, (ii) Scleroderma group, (iii) Mock group (empty microneedles without loading), (iv) HA@MS group (microneedle loaded with SRT1720@HA), (v) LNP@MS group (microneedle loaded with PGC1α@LNP), and (vi) HA/LNP@MS group (microneedle co‐loaded with SRT1720@HA and PGC1α@LNP).

Microneedle treatment was performed every other day for two weeks. At each session, the marked 1 cm × 1 cm dorsal fibrotic area was treated at five predefined insertion sites: the four corners and the center. For application, the proximal non‐threaded portion of each threaded microneedle was fixed in a sterile handheld microneedle holder to allow stable insertion and rotation. The microneedles were inserted perpendicular to the dorsal skin until the distal 1.5 mm drug‐loaded threaded region was fully embedded. After insertion, the microneedle was gently rotated, retained in the skin for 30 s, and then withdrawn. All animals were monitored throughout the study for signs of distress, skin ulceration, or infection, and appropriate care was provided as needed.

### 4.17 Histology, Immunohistochemistry, and Immunofluorescence

Tissue samples were fixed in 4% paraformaldehyde overnight, embedded in paraffin, and sectioned at 5 µm thickness. For histological evaluation, sections were stained with hematoxylin and eosin (H&E) using a standard protocol. Collagen deposition was assessed with Masson's Trichrome Stain Kit (Cat. No. G1340, Solarbio, Beijing, China), and elastic fiber distribution was examined using an Elastica van Gieson (EVG) staining kit (Cat. No. G1590, Solarbio, Beijing, China).

For immunohistochemistry, sections were deparaffinized, rehydrated, and subjected to antigen retrieval in citrate buffer at 95°C for 20 min. After blocking with 5% BSA, sections were incubated overnight at 4°C with primary antibodies against CDKN1A (CST, Cat. No. 2947, 1:200), CDKN2A (CST, Cat. No. 23200, 1:200), α‐SMA (CST, Cat. No. 19245S, 1:200), and COL1A1 (CST, Cat. No. 72026S, 1:200). Sections were then incubated with HRP‐conjugated Goat Anti‐Rabbit IgG (Jackson lab, Cat. No. 138729, 1:200) for 1 h at room temperature. Stained sections were imaged with a bright‐field microscope (Leica, Germany).

For immunofluorescence, sections were incubated overnight at 4°C with primary antibodies against PDGFRα (Santa Cruz, Cat. No. sc‐398206, 1:50) and CDKN2A (CST, Cat. No. 23200, 1:100), or with α‐SMA (CST, Cat. No. 19245S, 1:200) and Perilipin (Santa Cruz, Cat. No. sc‐390169, 1:50). After washing, sections were incubated with FITC‐conjugated Goat Anti‐Mouse IgG (Servicebio, Cat. No. GB22301, 1:200) and Cy3‐conjugated Goat Anti‐Rabbit IgG (Servicebio, Cat. No. GB21303, 1:200) for 1 h in the dark. Nuclei were counterstained with DAPI (Sigma, USA). Fluorescent images were acquired using a Nikon Eclipse E800 microscope (Nikon, Melville, NY, USA).

### 4.18 RNA Sequencing

For RNA‐Seq analysis, three biological replicates of full‐thickness skin tissue were collected per experimental group. Total RNA was extracted using TRIzol reagent (Invitrogen, Carlsbad, CA, USA), and RNA quality was assessed with the Agilent 2100 Bioanalyzer (Agilent Technologies, Santa Clara, CA, USA). Only samples with an RNA Integrity Number (RIN) ≥ 7 were used for library construction. cDNA libraries were prepared using the TruSeq Stranded mRNA LT Sample Prep Kit (Illumina, San Diego, CA, USA) and sequenced on the Illumina platform according to the manufacturer's protocol. Differentially expressed genes (DEGs) were identified using the R package DESeq2 (v1.6.3). Genes with *p* < 0.05 and a fold change > 2 or < 0.5 were considered significantly differentially expressed. Hierarchical clustering was applied to analyze expression patterns of DEGs. Gene Ontology (GO) enrichment and Kyoto Encyclopedia of Genes and Genomes (KEGG) pathway analyses were performed in R based on the hypergeometric distribution.

### 4.19 Spatial Transcriptomics Analysis

Spatial transcriptomic data were obtained from our previously published work [66] and reanalyzed to characterize layer‐specific molecular features of fibrotic skin. Raw count matrices and spatial spot information were downloaded from the original repository and processed using Seurat and SeuratData workflows in R. Low‐quality spots were removed based on mitochondrial gene percentage and total feature counts. Normalization, scaling, and dimensionality reduction were performed using the SCTransform pipeline. Spatial clustering was conducted with the FindNeighbors and FindClusters functions, and clusters were annotated according to marker genes reported in the original publication. Layer boundaries were determined based on spot coordinates and histological images provided by the dataset. Differentially expressed genes between layers and between experimental conditions were identified using FindMarkers with Wilcoxon rank‐sum tests. All analyses followed standard guidelines and were consistent with the procedures reported in the original study.

### 4.20 Statistical Analysis

All data are presented as mean ± standard deviation (SD). Statistical analyses were performed using Student's *t*‐test for comparisons between two groups and one‐way analysis of variance (ANOVA) followed by Dunnett's multiple‐comparisons test to compare each treatment group with the designated control group. A *p* value < 0.05 was considered statistically significant. All analyses were conducted using GraphPad Prism 8.0.

## Author Contributions

All authors reviewed and approved the final manuscript.

## Conflicts of Interest

The authors declare no competing interests.

## Supporting information




**Supporting File**: advs77624‐sup‐0001‐SuppMat.docx.

## Data Availability

The data that supports the findings of this study are available in the supplementary material of this article.
