# Supplementary material for: Layer‐Specific Dermal and Subcutaneous Delivery via Modular LNP–Hydrogel‐Integrated Threaded Microneedles
Source: Adv Sci (Weinh). 2026 Sep 21:e77624. Online ahead of print. doi: 10.1002/advs.77624 (PMC13591343; doi:10.1002/advs.77624)
Supplement: Supplementary file 1 [file ADVS-9999-e77624-s001.docx]

**Figure S1. In vitro release and functional stability of PGC1α@LNP.** A) Cumulative release profile of PGC1α mRNA from PGC1α@LNP over 96 h, showing sustained release with a cumulative release of 65% at 96 h. B) RT-qPCR analysis of PGC1α mRNA expression in 3T3-L1 cells following treatment with freshly prepared PGC1α@LNP(Fresh), lyophilized and immediately reconstituted PGC1α@LNP(Lyo-0M), or PGC1α@LNP reconstituted after one month of storage at −20°C(Lyo-1M). C) Western blot analysis of PGC1α protein expression in 3T3-L1 cells following treatment with the corresponding PGC1α@LNP formulations. *Statistical analyses were performed using one-way analysis of variance (ANOVA) followed by Dunnett’s multiple-comparisons test.*

**Figure S2. Concentration-dependent cytocompatibility of SRT1720@HA and PGC1α@LNP.** A) CCK-8 analysis of L929 cell viability after 24 h of incubation with SRT1720@HA hydrogel extracts at concentrations of 25%, 50%, 75%, and 100% (v/v). B) CCK-8 analysis of 3T3-L1 cell viability after 24 h of treatment with PGC1α@LNP at total lipid concentrations of 25, 50, 100, and 200 μg/mL. *Statistical analyses were performed using one-way analysis of variance (ANOVA) followed by Dunnett’s multiple-comparisons test.*

**Figure S3. Biodistribution and systemic exposure following local HA/LNP@MS administration.** A) RT-qPCR analysis of exogenous PGC1α mRNA in plasma, treated dorsal skin, draining lymph nodes, liver, and spleen at 1, 6, 24, and 48 h after HA/LNP@MS treatment. B) LC–MS/MS analysis of SRT1720 in the corresponding samples and time points.

**Figure S4. SRT1720@HA suppresses the secretion of IL-6 and IL-1β in senescent L929 fibroblasts.** A) ELISA quantification of IL-6 levels in the culture supernatants of L929 cells in the Control, Mock, HA, and SRT1720@HA groups. B) ELISA quantification of IL-1β levels in the corresponding culture supernatants. *Statistical analyses were performed using one-way analysis of variance (ANOVA), followed by Dunnett’s multiple-comparisons test.*

**Figure S5. Histological validation of the bleomycin-induced skin fibrosis model and the effect of uncoated microneedle insertion.** Groups: Normal, healthy mice without bleomycin or microneedle treatment; Scleroderma, mice with bleomycin-induced skin fibrosis without microneedle intervention; and Mock, fibrotic mice treated with uncoated microneedles. A) H&E staining of dorsal skin. B) Masson’s trichrome staining of dorsal skin, in which collagen fibers were stained blue, cytoplasm and muscle fibers were stained red. C) Immunohistochemical staining of α-SMA, with positively stained myofibroblasts appearing brown and indicated by black arrows. D) Immunohistochemical staining of COL1A1, with COL1A1-positive areas appearing brown. E) Quantification of dermal thickness corresponding to panel A. F) Quantification of subcutaneous fat layer thickness corresponding to panel A. G) Quantification of collagen-positive area corresponding to panel B. H) Quantification of α-SMA-positive cells corresponding to panel C. I) Quantification of the COL1A1-positive area corresponding to panel D. *Statistical analyses were performed using one-way analysis of variance (ANOVA), followed by Dunnett’s multiple-comparisons test.*

**Figure S6. RT-qPCR validation of representative differentially expressed genes identified by RNA sequencing.** Relative mRNA expression levels of Il6(A), Il1β(B), Lep(C), and Ppargc1α(D) in skin tissues from the Mock and HA/LNP@MS groups. *All scale bars are shown in the respective panels unless otherwise specified.* *Statistical analyses were performed using unpaired two-tailed t-tests (mean ± SD).*

**Table S1. Primers used for PCR in this study.**

| Primer | Sequences (5’-3’) |
| --- | --- |
| Gapdh | F：ACC ACA GTC CAT GCC ATC AC  R：TCC ACC ACC CTG TTG CTG TA |
| PPARγ | F：TTA TAG CTG TCA TTA TTC TCA GTG GAG  R：GAC TCT GGG TGA TTC AGC TTG |
| Fabp4 | F：TCG ACC ACA ATA AAG AGA AAA CG  R：CTT GTG GAA GTC ACG CCT TT |
| adiponectin | F：GGA GAG AAA GGA GAT GCA GGT  R：CTT TCC TGC CAG GGG TTC |
| EBPα | F：CCCTTGCTTTTTGCACCTCC  R：TGCCCCCATTCTCCATGAAC |
| Perilipine-1 | F：CTTGGGCGTCTGCCTTACCT  R：GCCCTTCAGTTCAGAGGCGA |
| Lipe | F：TGGGGTGACTCTAACGCGAC  R：AGGCCCCCAAACCATCCAAT |
| Col1A1 | F：GACAGGCGAACAAGGTGACAGAG  R：CAGGAGAACCAGGAGAACCAGGAG |
| α-SMA | F：CGTGGCTATTCCTTCGTGACTACTG  R：CGTCAGGCAGTTCGTAGCTCTTC |
| MMP9 | F：CAAAGACCTGAAAACCTCCAAC  R：GACTGCTTCTCTCCCATCATC |
| MCP1 | F：AAAACACGGGACGAGAAACCC  R：ACGGGAACCTTTATTAACCCCT |
| CDKN2A | F：CTTCGCCGAGCAGTTTCGT  R：TCAATCCCATCAGCCATTTCC |
| CDKN1A | F：CCCCCAATCGCAAGGATTCTT  R：CTTGGTTCGGTGGGTCTGTC |
| CCL5 | F：TTTGCCTACCTCTCCCTCG  R：CGACTGCAAGATTGGAGCACT |
| CCL10 | F：CACCATGAATCAAACTGCGA  R：GCTGATGCAGGTACAGCGT |
| IL6 | F：TGGGGCTCTTCAAAAGCTCC  R：AGGAACTATCACCGGATCTTCAA |
| IL1β | F：GAAATGCCACCTTTTGACAGTG  R：TGGATGCTCTCATCAGGACAG |
| Exogenous  PGC1α | F：TTTGACCCTGCTTCCACCAA  R：CGAGAGGCTTTTACCTGCGC |
| Ppargc1α | F：GGATGAAGACGGATTGCCCT  R：TCTGAGTGCTAAGACCGCTG |
| Lep | F：GTGCGGATTCTTGTGGCTTTGG  R：GAGACTGACTGCGTGTGTGAAATG |

**Table S2. mRNA sequences used in the study**

| mRNA | Sequences |
| --- | --- |
| mouse PGC1α | ATGTGCAGCCAAGACTCTGTATGGAGTGACATAGAGTGTGCTGCTCTGGTTGGTGAGGACCAGCCTCTTTGCCCAGATCTTCCTGAACTTGACCTTTCTGAACTTGATGTGAATGACTTGGATACAGACAGCTTTCTGGGTGGATTGAAGTGGTGTAGCGACCAATCGGAAATCATATCCAACCAGTACAACAATGAGCCTGCGAACATATTTGAGAAGATAGATGAAGAGAATGAGGCAAACTTGCTAGCGGTTCTCACAGAGACACTGGACAGTCTCCCCGTGGATGAAGACGGATTGCCCTCATTTGATGCACTGACAGATGGAGCCGTGACCACTGACAACGAGGCCAGTCCTTCCTCCATGCCTGACGGCACCCCTCCCCCTCAGGAGGCAGAAGAGCCGTCTCTACTTAAGAAGCTCTTACTGGCACCAGCCAACACTCAGCTCAGCTACAATGAATGCAGCGGTCTTAGCACTCAGAACCATGCAGCAAACCACACCCACAGGATCAGAACAAACCCTGCCATTGTTAAGACCGAGAATTCATGGAGCAATAAAGCGAAGAGCATTTGTCAACAGCAAAAGCCACAAAGACGTCCCTGCTCAGAGCTTCTCAAGTATCTGACCACAAACGATGACCCTCCTCACACCAAACCCACAGAAAACAGGAACAGCAGCAGAGACAAATGTGCTTCGAAAAAGAAGTCCCATACACAACCGCAGTCGCAACATGCTCAAGCCAAACCAACAACTTTATCTCTTCCTCTGACCCCAGAGTCACCAAATGACCCCAAGGGTTCCCCATTTGAGAACAAGACTATTGAGCGAACCTTAAGTGTGGAACTCTCTGGAACTGCAGGCCTAACTCCTCCCACAACTCCTCCTCATAAAGCCAACCAAGATAACCCTTTCAAGGCTTCGCCAAAGCTGAAGCCCTCTTGCAAGACCGTGGTGCCACCGCCAACCAAGAGGGCCCGGTACAGTGAGTGTTCTGGTACCCAAGGCAGCCACTCCACCAAGAAAGGGCCCGAGCAATCTGAGTTGTACGCACAACTCAGCAAGTCCTCAGGGCTCAGCCGAGGACACGAGGAAAGGAAGACTAAACGGCCCAGTCTCCGGCTGTTTGGTGACCATGACTACTGTCAGTCACTCAATTCCAAAACGGATATACTCATTAACATATCACAGGAGCTCCAAGACTCTAGACAACTAGACTTCAAAGATGCCTCCTGTGACTGGCAGGGGCACATCTGTTCTTCCACAGATTCAGGCCAGTGCTACCTGAGAGAGACTTTGGAGGCCAGCAAGCAGGTCTCTCCTTGCAGCACCAGAAAACAGCTCCAAGACCAGGAAATCCGAGCGGAGCTGAACAAGCACTTCGGTCATCCCTGTCAAGCTGTGTTTGACGACAAATCAGACAAGACCAGTGAACTAAGGGATGGCGACTTCAGTAATGAACAATTCTCCAAACTACCTGTGTTTATAAATTCAGGACTAGCCATGGATGGCCTATTTGATGACAGTGAAGATGAAAGTGATAAACTGAGCTACCCTTGGGATGGCACGCAGCCCTATTCATTGTTCGATGTGTCGCCTTCTTGCTCTTCCTTTAACTCTCCGTGTCGAGACTCAGTGTCACCACCGAAATCCTTATTTTCTCAAAGACCCCAAAGGATGCGCTCTCGTTCAAGATCCTTTTCTCGACACAGGTCGTGTTCCCGATCACCATATTCCAGGTCAAGATCAAGGTCCCCAGGCAGTAGATCCTCTTCAAGATCCTGTTACTACTATGAATCAAGCCACTACAGACACCGCACACACCGCAATTCTCCCTTGTATGTGAGATCACGTTCAAGGTCACCCTACAGCCGTAGGCCCAGGTACGACAGCTATGAAGCCTATGAGCACGAAAGGCTCAAGAGGGATGAATACCGCAAAGAGCACGAGAAGCGGGAGTCTGAAAGGGCCAAACAGAGAGAGAGGCAGAAGCAGAAAGCAATTGAAGAGCGCCGTGTGATTTACGTTGGTAAAATCAGACCTGACACAACGCGGACAGAATTGAGAGACCGCTTTGAAGTTTTTGGTGAAATTGAGGAATGCACCGTAAATCTGCGGGATGATGGAGACAGCTATGGTTTCATCACCTACCGTTACACCTGTGACGCTTTCGCTGCTCTTGAGAATGGATATACTTTACGCAGGTCGAACGAAACTGACTTCGAGCTGTACTTTTGTGGACGGAAGCAATTTTTCAAGTCTAACTATGCAGACCTAGATACCAACTCAGACGATTTTGACCCTGCTTCCACCAAGAGCAAGTATGACTCTCTGGATTTTGATAGTTTACTGAAGGAAGCTCAGAGAAGCTTGCGCAGGTAAAAGCCTCTCGTCGCAAGAATCTCATCCTGCACATCAATCC |
